# Direct Observation of Suppressed Optical–Acoustic Phonon Energy Coupling in Supported SWCNT at Cryogenic Temperatures

**DOI:** 10.1002/advs.202509005

**Published:** 2025-07-03

**Authors:** Ibrahim Al Keyyam, Yu Hua, Baini Li, Tianyu Wang, Cheng Deng, Xinwei Wang

**Affiliations:** ^1^ Department of Mechanical Engineering Iowa State University Ames IA 50011 USA; ^2^ Research Center for Industries of the Future Key Laboratory of 3D Micro/Nano Fabrication and Characterization of Zhejiang Province School of Engineering Westlake University Hangzhou 310024 P. R. China; ^3^ College of Mechatronics Engineering Guangdong Polytechnic Normal University Guangzhou 510635 P. R. China

**Keywords:** cryogenic phonon energy coupling, equivalent interfacial medium (EIM), FET‐Raman, intrinsic interfacial thermal conductance, nanoscale 1D material, phonon nonequilibrium

## Abstract

Nonequilibrium among phonon branches critically influences nanoscale heat transport yet remains largely unexplored in one‐dimensional (1D) systems, particularly at cryogenic temperatures. This work reports the first experimental quantification of optical–acoustic phonon coupling factor (*G*_OA_) in single‐walled carbon nanotubes using the frequency‐domain energy transport state‐resolved Raman technique at cryogenic and room temperatures. Remarkably, a strong suppression of *G*_OA_ is observed at low temperatures that exceeds the suppression of the coupling of interfacial phonon modes. As temperature increases, *G*_OA_ is found to increase monotonically, consistent with enhanced anharmonic decay processes of optical phonons. At 93 K, the optical–acoustic phonon temperature difference exceeds 75% of the acoustic phonon temperature rise, which is reduced to about 33% at room temperature. The critical role of laser heating size on phonon nonequilibrium is elucidated, where it gets amplified for a more confined heating size. By utilizing the recently developed equivalent interfacial medium model, the intrinsic temperature‐dependent interfacial thermal conductance based on acoustic phonon temperature is obtained. The results show that neglecting the nonequilibrium among phonon branches overestimates the interfacial conductance by ≈30% at room temperature. This research provides fundamental insights into phonon nonequilibrium in 1D nanoscale materials that strongly impact next‐generation nanoelectronics and solid‐state energy converters.

## Graphical abstract

This study quantifies the temperature‐dependent optical–acoustic phonon coupling factor (*G_OA_*) in supported single‐walled carbon nanotubes, revealing significant suppression at low temperatures. At high temperatures, *G_OA_* increases monotonically and is attributed to intensified anharmonic phonon interactions. This research offers key insights into phonon nonequilibrium in 1D nanoscale materials, with implications for future nanoelectronics and energy conversion technologies.

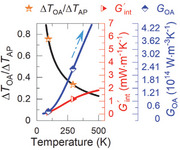

## 1 Introduction

Recent developments in nanoscale materials and devices are driving transformative advancements across electronics, optoelectronics, photonics, and thermoelectrics.^[^ ^1^, ^2^, ^3^ ^]^ At these scales, the efficiency of device operation significantly depends on energy carrier dynamics, primarily on how carriers dissipate energy through phonon interactions.^[^ ^4^ ^]^ When semiconductors are optically or electrically excited, carriers lose energy predominantly via phonon scattering processes, primarily involving optical phonons (OP) due to their strong electron coupling, before thermal energy dissipates through acoustic phonon (AP) transport.^[^ ^5^ ^]^ Under such conditions, inadequate phonon–phonon coupling often leads to distinct temperature distributions among different phonon branches, resulting in pronounced nonequilibrium effects.^[^ ^6^ ^]^ This phonon nonequilibrium can substantially impede heat dissipation, threatening device reliability, but can also be harnessed beneficially to prolong carrier lifetimes, thereby enhancing device performance in certain applications.^[^ ^7^, ^8^, ^9^, ^10^ ^]^ Hence, clarifying the fundamental mechanisms underlying phonon nonequilibrium is essential for future progress in nanoelectronics and energy technologies.

A great focus has been devoted to exploring thermal transport in nanoscale materials for its fundamental and engineering importance.^[^ ^11^ ^]^ Single‐walled carbon nanotubes (SWCNTs), owing to their extraordinary thermophysical properties,^[^ ^12^, ^13^, ^14^ ^]^ represent ideal candidates for highly efficient thermal management systems. Nonetheless, recent investigations reveal significant deviations from equilibrium phonon transport conditions in low‐dimensional materials under localized laser irradiation,^[^ ^15^, ^16^, ^17^, ^18^, ^19^ ^]^ which warrants a critical revisit of thermal transport in SWCNT. Resolving this phonon–phonon nonequilibrium is crucial for accurately characterizing the intrinsic thermal properties of SWCNTs and their coupling factors with other substrates, which is necessary to optimize their performance in nanoscale heat dissipation applications.^[^ ^20^ ^]^

While Raman‐based measurements have achieved significant success for thermal characterization,^[^ ^21^ ^]^ their effectiveness is inherently limited by their sole sensitivity to optical phonon temperatures, disregarding acoustic phonons which dominate thermal transport.^[^ ^22^ ^]^ As a result, traditional steady‐state Raman thermometry tends to overestimate the actual temperature rise, which yields an inaccurate determination of thermal transport properties of interest. Several studies indeed raised doubts about the accuracy of Raman thermometry, which mainly measures optical phonons temperatures.^[^ ^23^, ^24^ ^]^ If different phonon branches are not equilibrated, then the validity of Raman measurement must be rigorously addressed. First‐principle calculations on graphene by Bonini et al.^[^ ^25^ ^]^ are among the first to point out and quantify the nonequilibrium effect in graphene, but it remains an experimental challenge. To address this issue, Raman thermometry has to be combined with other types of spectroscopy, like the Brillouin light scattering, to measure the optical and acoustic temperatures separately.^[^ ^16^ ^]^

Alternatively, advanced Raman technologies such as frequency‐domain energy transport state‐resolved Raman (FET‐Raman) and nanosecond energy transport state‐resolved Raman have been developed and utilized to resolve optical/acoustic phonon temperatures separately.^[^ ^5^, ^18^, ^19^ ^]^ Wang et al.^[^ ^5^ ^]^ pioneered the experimental efforts toward distinguishing the acoustic–optical phonon temperatures in Raman measurements for 2D materials. Their results suggest that the OP–AP temperature difference accounted for over 30% of the Raman‐measured temperature rise for suspended MoS_2_ and MoSe_2_. They further quantify the energy coupling factors between phonon branches to fall in the range of 10^15^–10^16^ W m^−3^ K^−1^. A similar range has been reported for suspended WS_2_, where this temperature difference accounts for 37% of the acoustic phonon temperature, confirming the strong OP–AP thermal nonequilibrium.^[^ ^17^ ^]^ Interestingly, suspended graphene paper exhibits a substantially larger nonequilibrium, exceeding 80% of the acoustic phonon temperature.^[^ ^18^ ^]^ Most recently, it has been found that the nonequilibrium under a small laser spot size exceeds 120% of the AP temperature in supported MoS_2_.^[^ ^15^ ^]^

Despite the recent progress in addressing optical–acoustic phonon nonequilibrium, a research gap persists since existing literature has focused on phonon nonequilibrium at room temperature. However, as will be motivated shortly, the nature of the interactions between acoustic and optical phonons is highly temperature‐dependent. Hence, elucidating the phonon–phonon nonequilibrium at cryogenic temperatures is of huge interest. Further, existing literature has focused solely on 2D materials, leaving 1D materials like carbon nanotubes unexplored. The unique phonon confinement and dispersion in 1D systems and distinct scattering dynamics could lead to fundamentally different nonequilibrium effects.

This work uses the FET‐Raman technique to probe the frequency‐dependent transient thermal response of a nanometer thick SWCNT bundle supported on a SiO_2_ substrate at a controlled ambient temperature. A rigorous theoretical physical model is developed to detect the optical–acoustic phonon temperature difference and motivate its anticipated temperature dependence. The results are then utilized to infer the effective optical–acoustic phonon energy coupling factor *G_OA_* through a novel theoretical treatment, which we validate experimentally with previously reported data for 2D materials, and excellent quantitative agreement is established. The intrinsic interfacial thermal conductance between SWCNT and SiO_2_ substrate is measured based on the true acoustic phonon temperature, which we find to be overestimated if the phonon nonequilibrium is disregarded. We then utilize our recently developed universal model, the equivalent interfacial medium (EIM), to extend the observed results over a wider temperature range. A substantial suppression is observed for *G_OA_* at low temperatures that exceed the suppression of the coupling of acoustic interfacial phonon modes. We further elucidate the impact of the laser heating size on OP–AP nonequilibrium, where it diminishes for sufficiently large laser spot radii, consistent with first‐principle calculations and experimental data for 2D materials. These findings provide critical insights into phonon dynamics within supported samples and across interfaces, which strongly impact nanoscale thermal management strategies for next‐generation electronic and photonic devices.

The method developed here can potentially be adapted to characterize quantum dots (QDs) and other low‐dimensional materials (2D van der Waals crystals), provided the presence of a clearly detectable Raman‐active mode whose frequency shift accurately reflects temperature changes and distinguishable thermal responses between transient and steady‐state conditions. However, QDs pose substantial experimental challenges that limit the effectiveness of Raman‐based thermal characterization. Raman scattering from QDs is intrinsically weak, typically supplemented by enhancement methods such as surface‐enhanced Raman scattering^[^ ^26^ ^]^ and microcavities^[^ ^27^ ^]^ to obtain measurable signals. Additionally, the inherently strong fluorescence of QDs frequently dominates and obscures subtle Raman signals. These challenges emphasize why accurately characterizing interfacial thermal resistance and potential optical–acoustic phonon nonequilibrium of individual QDs through conventional Raman setups remains a significant challenge. On the other hand, ongoing efforts in our lab to test this method for 2D materials on different substrates are expected to be published in the near future.

## 2 Results and Discussion

### 2.1 FET‐Raman for Distinguishing OP–AP Thermal Nonequilibrium

**Figure 1:**
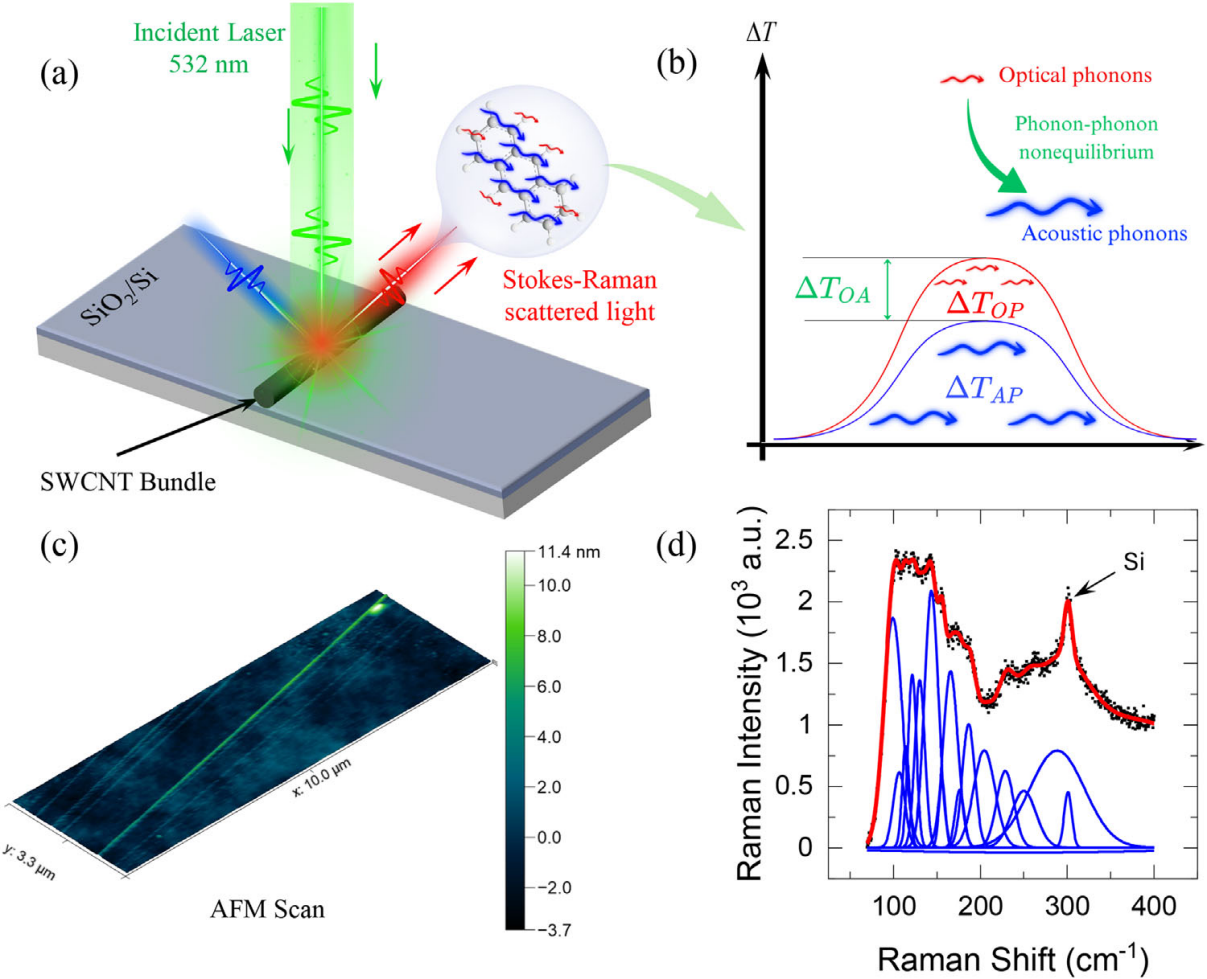
a) Schematic of the Raman experiment. A 532 nm laser irradiates the highly aligned SWCNT bundle supported on SiO_2_/Si substrate. The Stokes–Raman scattered light carries information about optical phonons under laser heating. b) A schematic diagram showing the optical–acoustic phonon temperature gradient under laser heating due to the OP–AP nonequilibrium. c) A 3D atomic force microscope (AFM) scan for sample #1 with the height shown on the scale to the right. The height is measured to be around 8 nm. d) The radial breathing modes (RBM), which are low‐frequency phonon modes in SWCNT. The RBM is fitted to multiple Gaussian functions to extract the unique frequencies in the spectrum.

**Figure 2:**
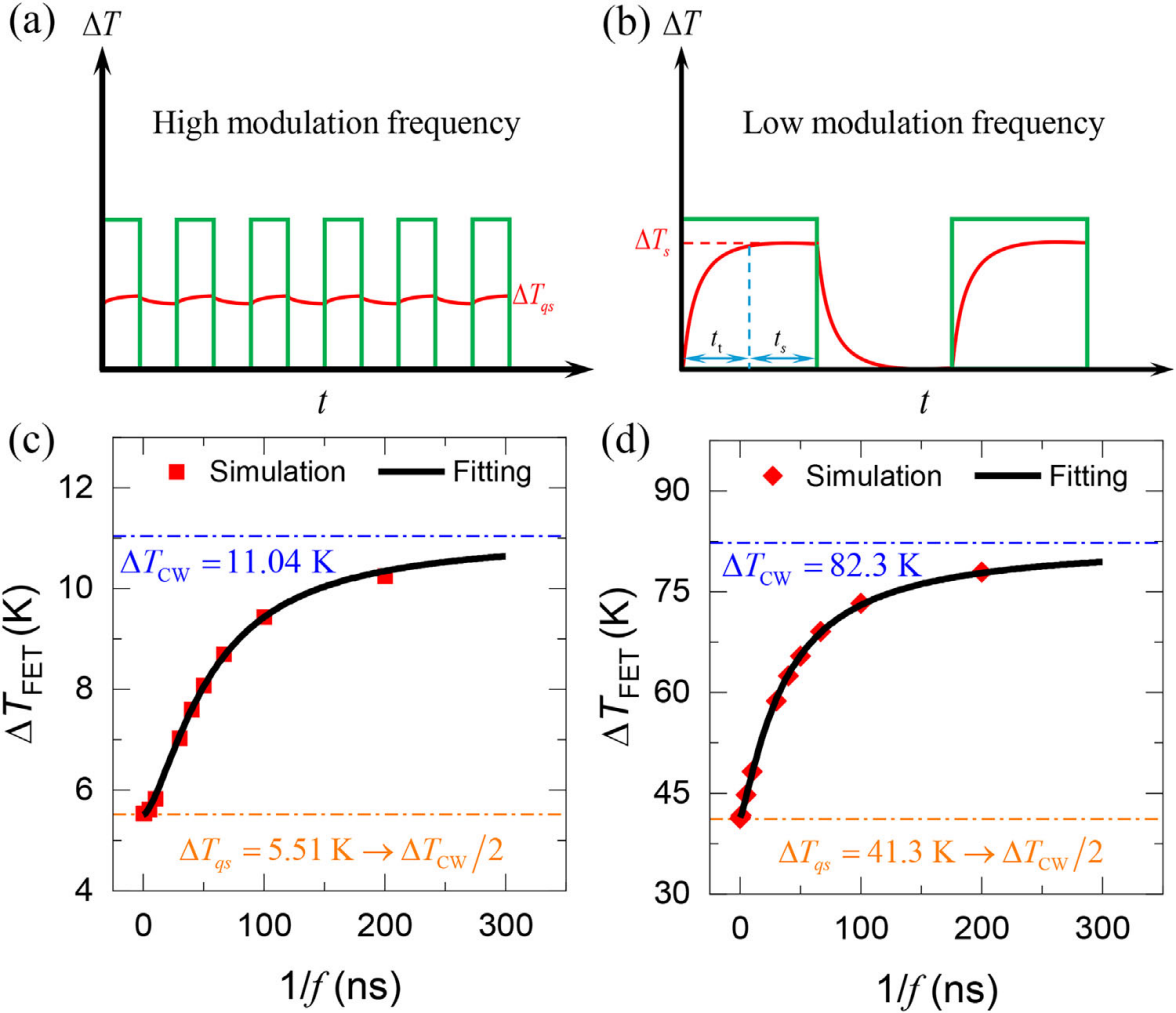
The physics of FET‐Raman under a) a high modulation frequency where the temperature rise for sufficiently high frequency approaches half of the steady‐state heating, and b) a low modulation frequency where the temperature rise approaches the steady‐state heating. Simulation results for the average temperature rise under the laser spot as a function of the pulse period under c) 20× and d) 100×. The temperature rise under the CW (∆*T*_CW_) laser is shown in blue dotted‐dashed line. The temperature rise at the infinite frequency limit (∆*T*_qs_) is shown to be half of that under a CW laser.

The schematic in **Figure** **1**a provides an overview of our FET‐Raman experiment, designed to probe phonon nonequilibrium in highly aligned supported‐SWCNTs on a 300 nm silicon dioxide layer on silicon substrate. A 532 nm laser beam excites the SWCNTs, causing them to emit scattered Stokes–Raman light, which is then collected for further analysis. The sample is enclosed in a temperature‐controlled stage (see Figure S1 in the Supporting Information), allowing precise ambient temperature control. Crucially, as illustrated, the SWCNTs exhibit two distinct phonon branches: acoustic phonons (indicated in blue) and optical phonons (indicated in red). Under laser heating, the phonon temperatures could deviate from equilibrium, as shown in **Figure** **2**b. Such nonequilibrium is driven by restricted energy exchange between acoustic and optical phonon branches, which will be detailed later in the paper. As shown in Figure 1c, sample #1 is characterized by using an atomic force microscope (AFM), which shows a high alignment for the bundle. The length of the sample is found to exceed 10 µm in length and is about 8 nm in height. We further analyze the SWCNT bundle's radial breathing mode (RBM) Raman spectrum. The spectrum exhibits several peaks, which we resolve using multiple Gaussian peak fittings (Figure 1d), each corresponding to a distinct RBM frequency (*ω*_RBM_). These frequencies directly correlate with specific SWCNT diameters *d* = 223.75/*ω*_RBM_, which can then be used to estimate the solid area of the SWCNT bundle, calculated as*A*_c_ = ∑(π/4)(*d*_o_^2^ − *d*_i_^2^), where *d*_o_ is the diameter of individual SWCNTs as revealed by the RBM, *d*_i_ = *d*_o_ − 2*t*, and *t* = 0.335 nm as the SWCNT wall thickness.^[^ ^28^ ^]^ Further detailed discussion is outlined in earlier published work.^[^ ^29^, ^30^ ^]^

To motivate our experimental method, we show the difference in the thermal response of the sample under a modulated laser with different modulation frequencies in Figure 2. When the modulation frequency is very high (Figure 2a), the sample lacks the time to heat up or cool down fully within each modulation cycle. This creates a quasi‐steady‐state condition, where the average temperature rise under the laser spot area approaches precisely half the value observed under continuous wave (CW) laser heating. As the heating time increases, the sample has enough time to respond, and its average temperature starts to increase until it approaches the CW laser heating for sufficiently low modulation frequencies (Figure 2b).

To verify this argument, we solve the heat conduction equation of a supported SWCNT bundle under laser heating for steady and transient states described as

(1) $$k\frac{\partial^{2}T}{\partial x^{2}}-\frac{T-T_{s}}{R^{\prime }\cdot A_{c}}+\dot{q}=\rho c\frac{\partial T}{\partial t^{\cdot }}$$

The transient term on the right‐hand side is set to zero for steady‐state heating. Here, *κ* denotes the thermal conductivity of the sample as measured previously in our lab,^[^ ^31^ ^]^ *R*′ is the interfacial thermal resistance, *ρ* is the density, *c* is the specific heat capacity, *T*_s_ ​is the temperature of the substrate, which is held constant as demonstrated in our previous works,^[^ ^29^, ^30^ ^]^ and *A*_c_ is the solid cross‐sectional area of the SWCNT bundle. The simulation input parameters are chosen based on previous measurements and are summarized in **Table** **1**. The induced laser heating ($\dot{q}$) is represented by a Gaussian profile and is defined as

(2) $$\dot{q}\left(x\right)={\dot{q}}_{0}\exp\left(-x^{2}/x_{0}^{2}\right)$$

where ${\dot{q}}_{0}$ is the peak heat source at the center of the laser beam (*x* = 0) and is taken to be 1 mW for sole modeling purpose, and *x*_0_ is the radius of the laser beam. Since the measured temperature rise during the Raman experiment (*ψ*) (see the Experimental Section) is the average under the laser spot area, the theoretical temperature rise from solving the heat equation must be averaged. For steady‐state heating, the Raman‐intensity weighted average temperature rise can be calculated by integrating over the spatial domain as $\Delta T_{CW}=\int_{0}^{x_{0}}I\Delta T\cdot dx/\int_{0}^{x_{0}}I\cdot dx$. For the transient state heating, since the temperature is time‐dependent, the temperature rise must be averaged over both temporal and spatial domains as $\Delta T_{FET}=\int_{0}^{t_{0}}\int_{0}^{x_{0}}I\Delta T\cdot dxdt/\int_{0}^{t_{0}}\int_{0}^{x_{0}}Idxdt$, where *t*_0_ is the laser heating time (0.5/*f*). In both cases, *I* is the intensity taken as $I=I_{0}\exp\left(-x^{2}/x_{0}^{2}\right)$, where *x* is the distance from the laser center.

**Table 1:** Simulation input parameters.

Parameter	Value
Sample length [µm]	100
Sample diameter [nm]	6
Thermal conductivity [W m^−1^ K^−1^]^[^ ^29^ ^]^	200
Volumetric heat capacity [MJ m^−^^3^ K^−1^]^[^ ^31^ ^]^	1.36
Radius of laser spot [µm]^[^ ^29^ ^]^	0.4–1.8
Interfacial thermal resistance [K m W^−1^]^[^ ^29^ ^]^	1000
Number of grid points	3000
Laser modulation frequency [MHz]	5–5000

The theoretical model above is solved using our recently developed ultrafast numerical method that utilizes complex modeling with Fourier transform. The results match the ones from a finite‐difference method with less than 1% discrepancy and about two orders of magnitude less computational cost. As clearly shown in Figure 2c,d for 20× and 100× objective lenses (laser spot radius of 1.8 and 0.4 µm, respectively), the quasi‐steady‐state temperature at the high‐frequency limit (1/*f* → 0) approaches half under CW heating. For the data fitting, we tested several functional forms under different simulation conditions, including variations in sample size, thermophysical properties, and laser spot radius. The results show that $\Delta T\left(f\right)=\Delta T_{\mathrm{CW}}-A/\left[{\left(f_{0}/f\right)}^{n}+1\right]$ yields the best fit, especially in the high‐frequency regime where other functions fail. Here, *A*, *f*_0_, and *n* are all constants to be determined by fitting. We note that this functional form is not motivated by the physics of heat transport and is solely used for its superior fitting capability as opposed to single‐exponential lumped analysis, double‐exponential models, or other physically motivated functions. Figure 2c,d shows the fitting using this function, and an excellent fitting is observed. The temperature rises which determined at the limit of 1/*f* → 0 as shown are equal to the half temperature rise under CW laser heating within the fitting uncertainty.

In the Raman experiment, the average temperature rise is analyzed through what we term as the Raman shift power coefficient calculated as *ψ* = ∂ω/∂*P* which is proportional to the temperature rise under the laser spot area (see the Experimental Section). Despite that the Raman spectrum carries information about the sample and the substrate, the use of the *G* band which is a characteristic feature of the SWCNT Raman modes prevents any misinterpretation due to signal overlapping from the substrate. We have shown in earlier work^[^ ^30^ ^]^ that the substrate contribution to heat transfer is minimal and that the thermal transport is mainly governed by the coupling between the SWCNT and the substrate. As such, no further analysis is needed for the thermal transport in the substrate. As will be detailed in Section 2.2, the temperature rise measured in the Raman experiment is the optical phonon temperature, which need not necessarily be the same as the acoustic phonon temperature. The above simulation, on the other hand, considers that acoustic phonons dominate heat conduction and neglects potential nonequilibrium between acoustic and optical phonons. For sufficiently thermalized phonons, optical and acoustic phonons should share the same temperature. Therefore, the experimental average temperature rise at infinite frequency (ψ_0_) is expected to be half the CW temperature rise (ψ_*CW*_) in the absence of OP–AP thermal nonequilibrium. Hence, by inspecting the temperature rise at the infinite frequency limit, one can detect the nonequilibrium if ψ_0_ exceeds 0.5ψ_*CW*_, which can only be associated with optical phonons since acoustic phonons cannot exceed this limit, as demonstrated in our simulations and physical development. Thus, we can quantify the degree of the OP–AP thermal nonequilibrium as ψ_*OA*_ = 2ψ_0_ − ψ_*CW*_. One can see that ψ_*OA*_ goes to zero (i.e., nonequilibrium vanishes) when ψ_0_ is equal to 0.5ψ_*CW*_ which matches the theoretical construction as established earlier. By quantifying this deviation, the experiment can accurately determine the extent of nonequilibrium and thus directly measure the temperature difference between acoustic and optical phonons. This, however, is insufficient to quantify the coupling between the two phonon branches, which will be detailed in Section 2.3 later in the paper.

**Figure 3:**
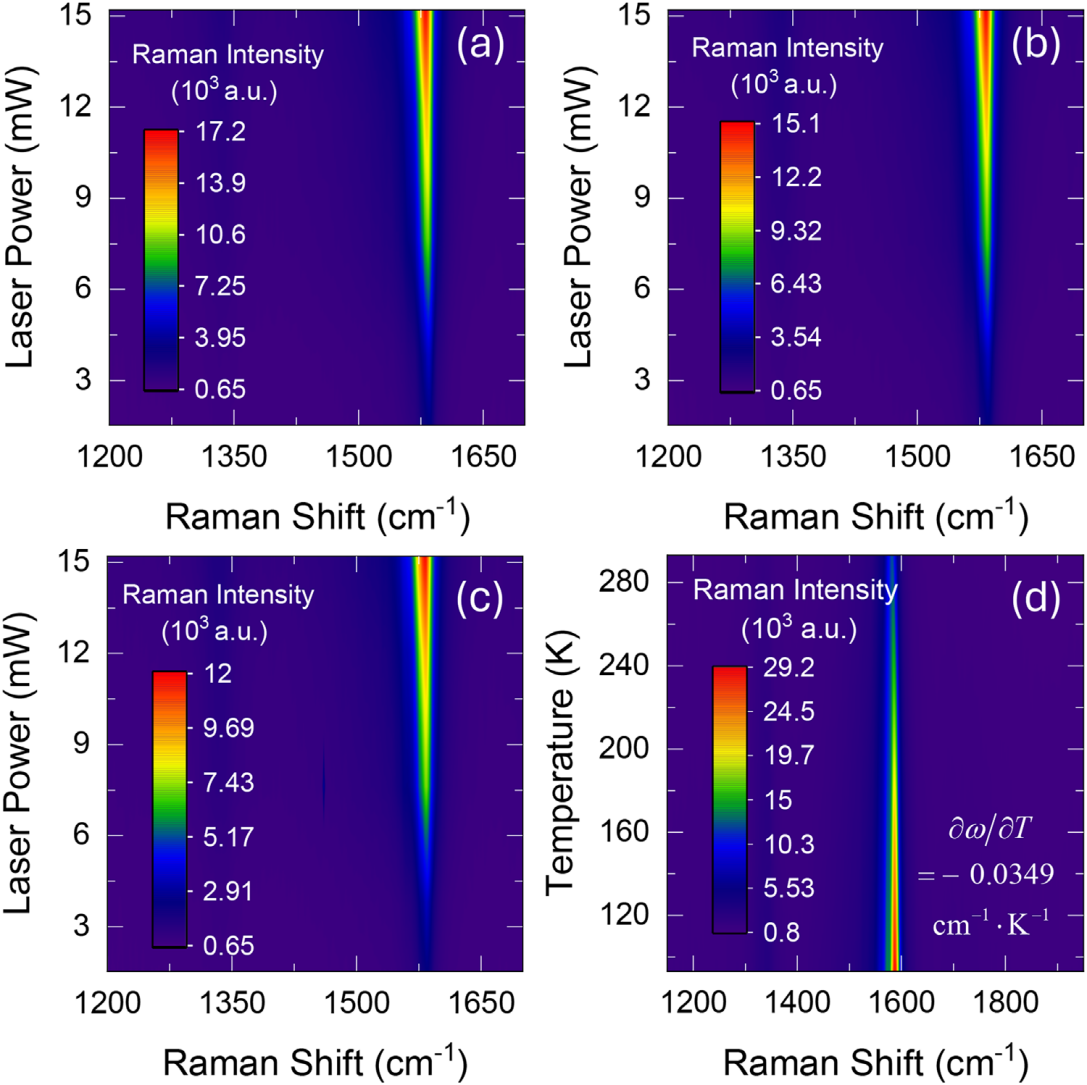
The Raman intensity and wavenumber variation with laser power under 50× objective lens for a) CW laser heating, b) 5 MHz modulated laser, c) 25 MHz modulated laser. d) Raman spectrum contour from 93 to 293 K. The Raman temperature coefficient is measured to be −0.0349 cm^−1^ K^−1^, in line with a previous measurement of −0.031 cm^−1^ K^−1^ for SWCNT.^[^ ^31^ ^]^

**Figure 4:**
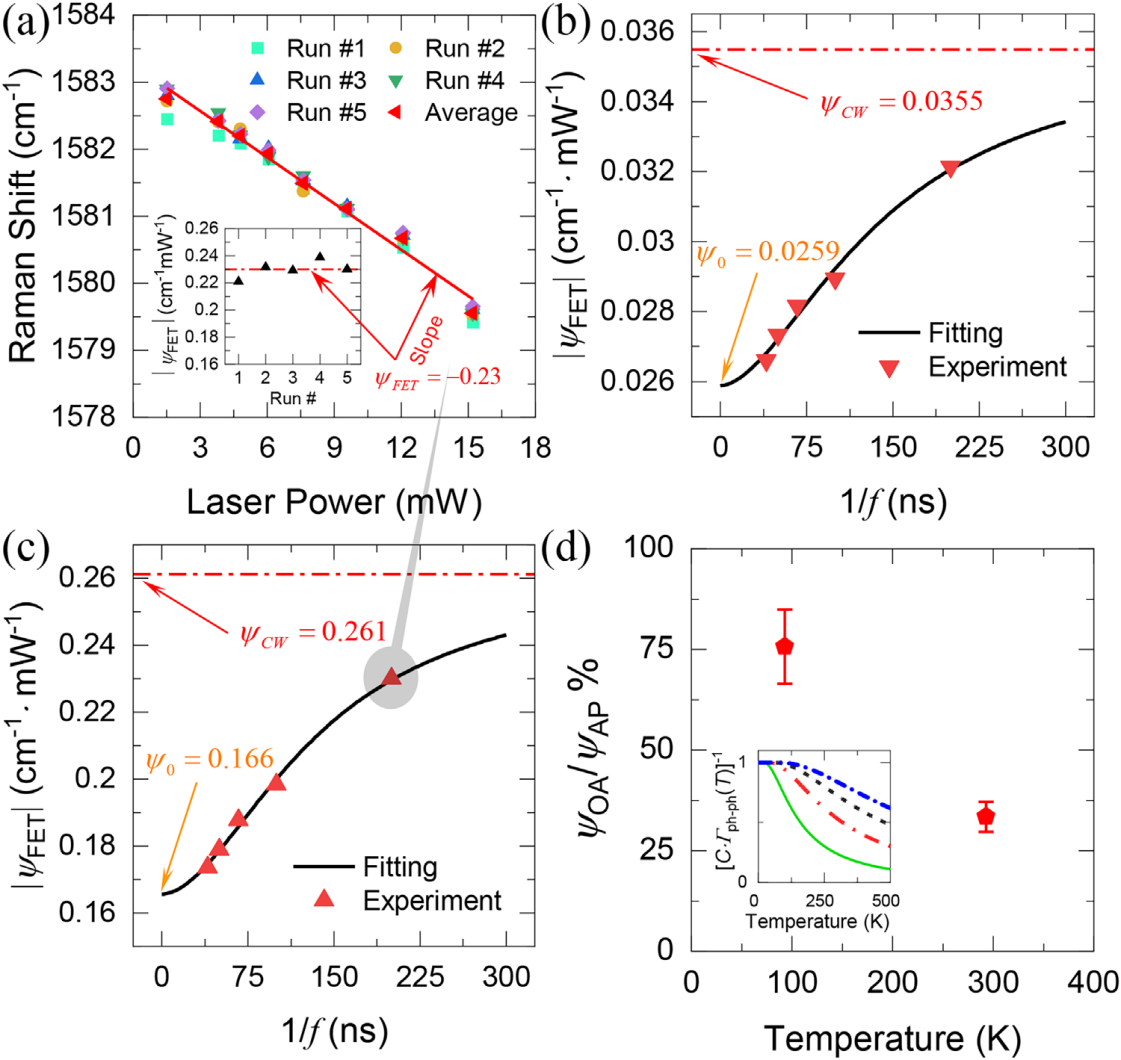
a) The Raman shift variation with laser power under 5 MHz modulated laser heating. The experiment is repeated over several runs and averaged to minimize the experimental errors. The inset shows the absolute value of the Raman shift power coefficient |ψ_*FET*_| (i.e., the slope of the data) for different runs and their average. b) |ψ_*FET*_| for different laser modulation frequencies at 93 K. c) |ψ_*FET*_|at 293 K. The dotted‐dashed lines are the Raman shift power coefficient under a CW laser |ψ_*CW*_|. d) The OP–AP temperature difference relative to the AP temperature rise as a function of ambient temperature. The inset shows the inverse of the phonon decay rate (phonon lifetime), discussed in the next section (see Figure 5b for the phonon decay rate), which resembles the trend observed in temperature‐dependentΔ*T_OA_*/Δ*T_AP_*.

To apply our method experimentally, we first obtain the steady state ψ_*CW*_ by conducting the experiment (detailed in the Experimental Section) at 293 K ambient temperature and CW laser. To obtain ψ_0_, we repeat the same experimental procedure to measure ψ_*FET*_ at five different frequencies: 5, 10, 15, 20, and 25 MHz. We then use the same fitting function motivated earlier $\psi_{\mathrm{FET}}\left(f\right)=\psi_{\mathrm{CW}}-A/\left[{\left(f_{0}/f\right)}^{n}+1\right]$ to obtain ψ_0_ at the infinite frequency limit. It is understandable that the higher the modulation frequency limit, the better the accuracy of the extrapolation. Although the function generator can exceed the frequency range used in this study, the electro‐optic modulator has a response limit of 25 MHz. Yet, we ensure that the current frequency range is sufficient to capture half the steady state temperature rise at the infinite frequency limit when extrapolating the simulation results. Selected results of ψ_*FET*_ and ψ_*CW*_ are illustrated in **Figure** **3**a–c, showing the Raman intensity and wavenumber variations under different laser power values at room temperature. A clear redshift of the G band is observed as laser power increases, which we quantify using ψ. The entire experimental procedure is also repeated at 93 K to obtain ψ_*FET*_, ψ_*CW*_ (see **Figure** **4**b), and hence ψ_0_ which we will discuss shortly. The choice of the cryogenic temperature was somewhat constrained by the temperature stage capability, despite that it can go a little below 93 K. However, the cell temperature can indeed go well beyond room temperature and up to 600 °C. We note that conducting the Raman experiments successfully at high temperature is also limited by getting a sound Raman signal which usually gets more challenging as you ramp up the temperature. It is of great interest to do a systematic parametric study and investigate the detailed temperature effect over a wide temperature range and could be considered in future work. Figure 3d provides the Raman temperature coefficient by linearizing the G band Raman shift ω_G_ between 93 and 293 K. The obtained temperature coefficient −0.0349 cm^−1^ K^−1^ closely aligns with a previously reported value −0.031 cm^−1^ K^−1^ for suspended SWCNT,^[^ ^31^ ^]^ which validates our experimental temperature measurements.

The measured ψ_*FET*_ at 93 and 293 K at different frequencies are shown in Figure 4b,c, respectively. The CW laser heating measurements ψ_*CW*_ are shown as dotted‐dashed lines. As we illustrate in Figure 4a, the experiment is repeated multiple times at each frequency to ensure the repeatability and reliability of the measured ψ_*FET*_ and to average out experimental uncertainties. The same fitting function, validated in the simulation part earlier, is used to fit the experimentally measured data to extract ψ_0_ at the infinite‐frequency limit. The ψ_*OA*_ is then determined using the same equation established earlier (ψ_OA_ = 2ψ_0_ − ψ_CW_). The acoustic phonon temperature rise effect is then determined as ψ_*AP*_ = ψ_*OP*_ − ψ_*OA*_. The results plotted in Figure 4d show that at 93 K, ψ_*OA*_ can exceed 75% of the acoustic phonon temperature rise (ψ_*AP*_). Interestingly, we find that the degree of nonequilibrium is suppressed as temperature rises, which we will explain by the enhanced phonon–phonon interaction discussed earlier and will be detailed shortly.

### 2.2 Temperature Dependence of Phonon–Phonon Interactions

As mentioned earlier, the primary heat carriers in SWCNTs are categorized into optical phonons and acoustic phonons. Optical phonons, which have high energy and low group velocity, primarily govern energy exchange mechanisms^[^ ^32^ ^]^ while acoustic phonons dominate heat conduction^[^ ^29^ ^]^ due to their higher lifetimes and group velocities. Under localized heating, absorbed energy initially excites hot electrons that thermalize quickly through fast electron–electron scattering,^[^ ^33^, ^34^ ^]^ which then transfer energy to optical phonons via electron–phonon interactions. This energy is cascaded to acoustic phonons through phonon–phonon scattering.^[^ ^5^ ^]^ However, because optical and acoustic phonons have different relaxation times, a measurable temperature difference between these branches emerges, leading to OP–AP thermal nonequilibrium. Recent studies have demonstrated that the OP–AP temperature difference (Δ*T*_OA_) can account for a substantial portion of the lattice temperature rise in nanomaterials, making it crucial to quantify this effect to accurately measure thermal properties.

The interaction between optical and acoustic phonons plays a fundamental role in dictating the thermal transport in low‐dimensional materials. Optical phonons undergo anharmonic decay into lower‐energy acoustic phonons before dissipating into the substrate.^[^ ^35^ ^]^ First‐principle studies have suggested a persistent nonequilibrium between optical and acoustic phonons upon perturbation.^[^ ^36^, ^37^ ^]^ The nonequilibrium between electrons and phonons in metallic thin films under ultrafast laser heating has been well established theoretically^[^ ^38^, ^39^, ^40^ ^]^ and resolved experimentally^[^ ^41^ ^]^ where the coupling factor between electrons and phonons is measured. An analogous treatment has been proposed in our group, and a coupling factor between different phonon branches is experimentally detected in 2D materials.^[^ ^5^, ^6^, ^15^, ^18^, ^19^ ^]^

Understanding the temperature dependence of this nonequilibrium and its governing mechanisms remains a very challenging subject of interest. The decay of optical phonons into acoustic modes strongly depends on temperature through anharmonic phonon–phonon interactions. Experimental Raman spectroscopy studies have consistently shown that optical phonon lifetimes shorten as temperature increases, which is evident through linewidth broadening.^[^ ^42^ ^]^ Theoretical and experimental studies have demonstrated that this decay rate exhibits strong temperature dependence, with different scaling behaviors in low‐ and high‐temperature regions.^[^ ^24^, ^43^ ^]^ Ultrafast pump–probe measurements^[^ ^44^ ^]^ provided direct evidence, showing the G‐mode phonon decay rate in SWCNTs nearly doubles from 300 to 700 K, while graphite displays a smaller but measurable increase. The stronger temperature dependence of SWCNT than graphene is likely due to additional decay pathways involving nanotube‐specific low‐frequency modes, such as radial breathing or bending modes. These extra channels accelerate phonon decay beyond standard predictions. Studies on electrically biased CNTs^[^ ^45^ ^]^ further reaffirm that the phonon decay bottleneck can be alleviated by increasing the lattice temperature, strengthening anharmonic interactions, and facilitating optical phonon relaxation into acoustic modes.

**Figure 5:**
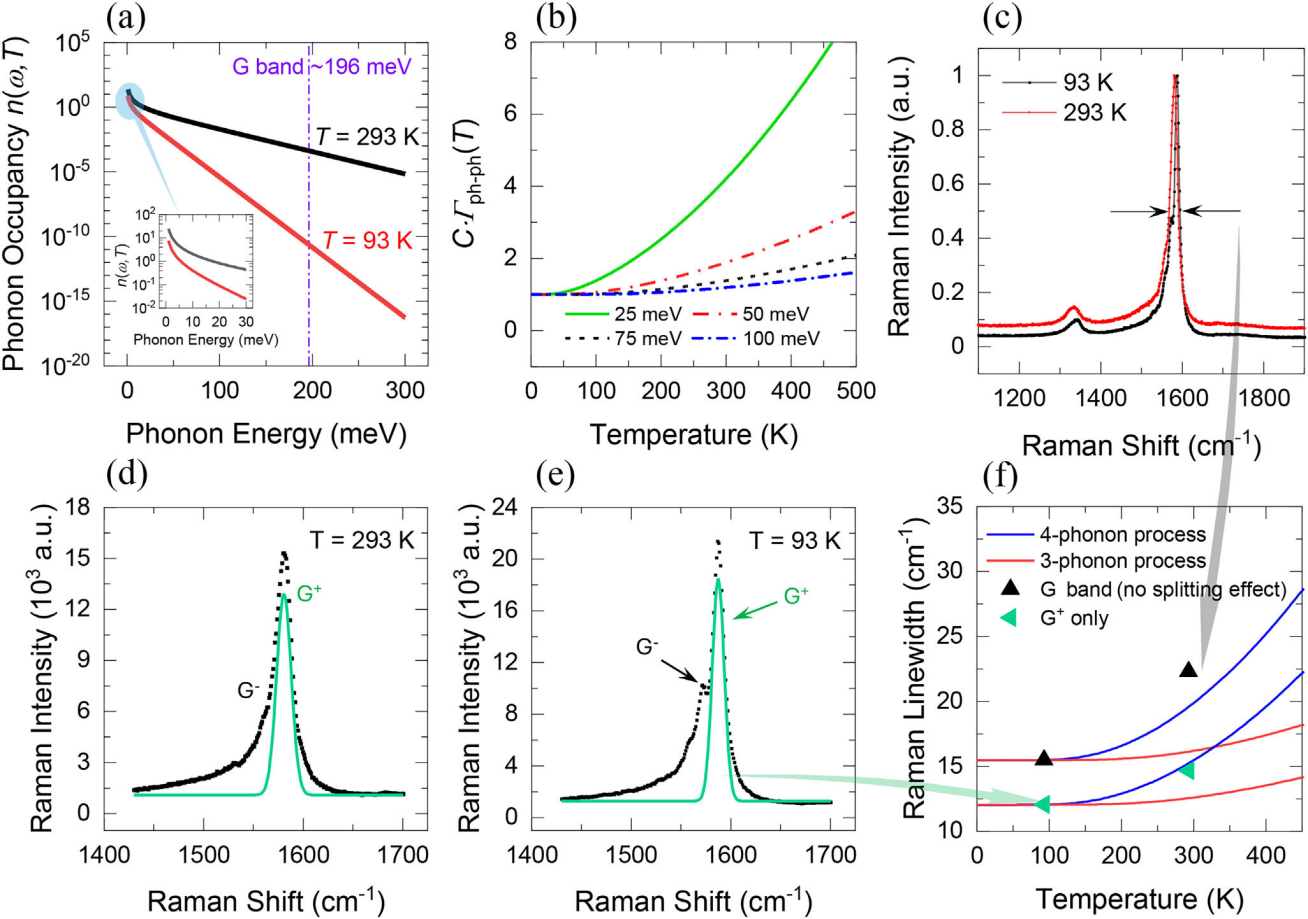
a) The phonon occupancy as a function of phonon energy and temperatures of interest. b) Normalized three‐phonon decay process as a function of temperature for different phonon energies. c) The normalized Raman spectrum of the sample of interest at 93 and 293 K with the G band linewidth highlighted. The G^+^ phonon mode fitted using the Gaussian function at d) 293 K and e) 93 K. f) The extracted linewidth for the G/G^+^ as a function of temperature. The experimental data are fitted using the 3‐ and 4‐phonon decay theories.

The phonon population exhibits strong temperature dependence that spans orders of magnitude, as shown in **Figure** **5**a, even for acoustic phonons in the low‐frequency range shown in the inset. The G band, an optical phonon mode in carbon nanotubes with an energy of ≈196 meV, is shown in a vertical dotted‐dashed line. The population is shown at two temperatures, 93 and 293 K, by using the Bose–Einstein statistics governed by $n\left(\omega ,T\right)=1/\left(\exp\left(\hbar \omega /k_{B}T\right)-1\right)$. The lowest anharmonic decay is a three‐phonon process known as the Klemens process,^[^ ^46^ ^]^ where one optical phonon decays into two lower‐energy phonons that conserve energy and momentum. This process is described by Γ(*T*) = Γ_0_[1 + *n*(ω_1_,*T*) + *n*(ω_2_,*T*)] where *n*(ω, *T*) is the occupation of a given phonon mode, and Γ_0_ is the decay rate at 0 K attributed to the decay due to the structural quality of the sample of interest and denotes phonon scattering mechanisms arising from defects, grain boundaries, impurities, or inhomogeneities in charge carrier density.^[^ ^47^ ^]^ Interestingly, we believe this has an intimate relationship to residual thermal reffusivity theory^[^ ^48^ ^]^ developed in our lab that quantifies the average structural thermal domain size from macroscopic quantities, namely thermal conductivity and volumetric heat capacity. Exploring the relationship between the two concepts and bridging them would be of enormous interest, but it is beyond the scope of this study and could be a topic of future research endeavors.

Figure 5b shows the temperature dependence of the 3‐phonon process for different phonon energies normalized to the decay rate at 0 K. The decay rate increases with temperature due to higher phonon populations, as shown in Figure 5a, enhancing the availability of these phonons and increasing the interaction probability. As shown, at given finite temperature, the decay of low‐energy phonons is more probable due to their larger occupancy. The G band inspected in this work, however, has a higher energy (≈196 meV) than the range plotted in Figure 5b. As will be discussed shortly, we find the 3‐phonon process is insufficient to explain the temperature‐dependent increase of the decay rate of the G band as observed in the Raman measurement. In the inset of Figure 4d, we show the inverse of the decay rate (lifetime), which we find to resemble the nonequilibrium temperature difference ratio, as will be elaborated on in Section 2.3. Overall, it is evident that at low temperatures, one shall expect a higher phonon–phonon nonequilibrium due to their limited interactions.

The linewidth of Raman bands is closely associated with the decay rate of carriers, primarily due to phonon–phonon interactions, where it indicates the lifetime and relaxation processes of optically excited carriers, as broader linewidths correspond to faster decay rates.^[^ ^25^ ^]^ The linewidth increases with temperature due to enhanced phonon–phonon scattering, particularly from four‐phonon processes, which were previously underestimated in theoretical models. The contribution of electron–phonon (e–ph) coupling to Raman linewidth in graphene and related materials is found to be nonmonotonic with temperature and, in most experimentally relevant regimes, acts to narrow the Raman peak rather than broaden it as shown in literature.^[^ ^49^ ^]^ In our measurements (Figure 5f), the observed linewidths increase markedly with temperature, reaching values that already match or surpass the predictions from four‐phonon decay models when G band splitting is neglected. Incorporating e–ph interactions into these calculations would further reduce the calculated linewidths, thereby increasing the discrepancy between theory and experiment. Consequently, we conclude that e–ph coupling plays a negligible role in accounting for the experimentally observed temperature dependence of the linewidth. Figure 5c shows the Raman spectra of the G band in our sample at 93 and 293 K, with clear broadening at the higher temperature. This broadening indicates a shorter phonon lifetime, implying a higher decay rate consistent with previous theoretical arguments. Figure 5f illustrates the extracted linewidth (shown in black triangles) against temperature and the fitting using three‐ and four‐phonon decay. We find the fitting to be in a better agreement with the four‐phonon decay, where it seems to dominate the interactions, which confirms their significant role even at room temperature, as suggested by Ruan and co‐workers.^[^ ^49^, ^50^ ^]^

Despite that, we find the linewidth broadening to exceed the theoretical prediction, even for the 4‐phonon process. A plausible reason behind the broadening as a function of temperature is the observed splitting of the G band, a well‐known feature of SWCNT. The splitting of the G band in suspended graphene has been observed and attributed to the mismatch in thermal expansion coefficient between graphene and the SiO_2_/Si substrate that enhances anisotropic strain in the graphene monolayer.^[^ ^51^ ^]^ We consequently isolate the G^+^ mode (see Figure 5d,e) and refit the results to 3‐ and 4‐phonon decay theories (see Figure 5f shown in light green triangles) and a better agreement is observed, suggesting that the splitting has a measurable effect when interpreting the linewidth measurements. The lifetime of the G^+^ mode is estimated through the energy–time uncertainty to be 0.44 and 0.36 ps at 93 and 293 K, respectively.^[^ ^52^ ^]^ We find the results to be slightly shorter than the reported values of 0.588–1.25 ps for SWCNT above 300 K.^[^ ^44^ ^]^ It is plausible that the broadening measured and shorter lifetime inferred is due to the bundling effect^[^ ^53^ ^]^ as well as any instrument broadening that we have not accounted for in the previous calculation. Another plausible reason is the structural defects as elaborated on in a previous work in our lab,^[^ ^31^ ^]^ which impacts the intrinsic linewidth Γ_0_. Overall, the above results confirm the faster optical decay rate as the temperature increases, which yields a higher phonon–phonon energy coupling and, thus, a lower OP–AP temperature difference, as observed earlier in Figure 4d and will be detailed shortly to quantify the coupling factor.

### 2.3 OP–AP Energy Coupling and Intrinsic Interfacial Thermal Conductance

The optical–acoustic phonon energy coupling factor (*G_OA_*) quantifies how efficiently energy transfers from optical phonons to acoustic phonons within the material. For supported samples under external excitation, energy is absorbed by the sample and then transferred to the substrate through interfacial thermal conductance (or thermal boundary conductance).^[^ ^54^ ^]^ Several studies show no direct coupling of optical phonons in the sample to phonons in the substrate, and the interfacial thermal conductance is mainly sustained via acoustic phonons.^[^ ^55^ ^]^ Hence, at (quasi) steady‐state, the energy transferred from optical phonons to acoustic phonons across the sample's volume must equal the energy dissipated from acoustic phonons to the substrate through the interface. Experimentally, to derive *G_OA_* for supported samples​, we start from the energy balance between optical and acoustic phonons, which can be described as $G_{OA}V\Delta T_{OA}=G_{int}^{\prime }L\Delta T_{AP}$, where Δ*T_OA_* is the OP–AP temperature difference, Δ*T_AP_* is the acoustic phonon temperature rise, and *V* and *L* are the volume and length of the sample, respectively. The $G_{int}^{\prime }$ (= 1/*R*′) is the interfacial thermal conductance with the substrate per unit length as measured in our experiment and will be elaborated on shortly. Manipulating the previous equation yields what we denote as *ξ* = Δ*T_OA_*/Δ*T_AP_* = *G*′_*int*_/(*G_OA_* · *A_c_*), where *A*_c_ is the cross‐sectional solid area of the SWCNT bundle as obtained previously. Finally, to obtain the *G*_OA_ from *ξ*, we must determine the interfacial thermal conductance $G_{int}^{\prime }$. Note here the above equation neglects the thermal nonequilibrium within acoustic phonon branches and optical phonon branches. Therefore, our evaluation of *G_OA_* should be treated as first‐order estimation, and this will not alter the conclusions drawn in the work.

**Figure 6:**
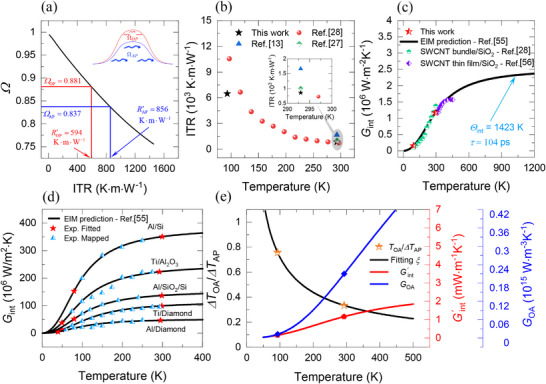
a) The heat conduction simulation results for the transient average temperature rise relative to the steady state one (Ω = ψ_*FET*_/ψ_*CW*_) as a function of the interfacial thermal resistance (ITR) at 5 MHz modulation frequency. The measured Ω_*OP*_ and Ω_*AP*_ are mapped out to infer the ITR (reciprocal of $G_{int}^{\prime }$). b) Measured ITR and its comparison with the previous measurements in the literature of supported SWCNT. c) The equivalent interfacial medium (EIM) model prediction of $G_{int}$ based on the two experimental measurements (for assumed 1 nm contact width) as a function of temperature. The graph shows the interface characteristic temperature (*Θ*_int_​) and the energy carrier transfer time (*τ*). Reported measurements in the literature are mapped onto the graph, showing great quantitative agreement with the EIM prediction. d) Validation of two‐measurement fitting using the EIM model for various interfaces in literature adapted from ref. [57] in the original work. Solid red stars are measurements used as inputs to the EIM fitting, whereas blue‐white triangles are mapped onto the prediction. The remarkable agreement validates the use of the EIM model. e) The measured Δ*T*_OA_/Δ*T*_AP_ plotted on the left *y*‐axis fitted to *ξ*. The EIM prediction of $G_{int}^{\prime }$ is shown on the first right *y*‐axis in red. The inferred *G_OA_* is shown on the far‐right *y*‐axis in blue.

**Figure 7:**
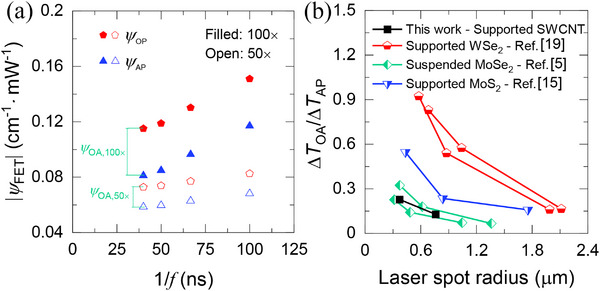
a) The measured ψ_*OP*_ and extracted ψ_*AP*_ along with their temperature difference ψ_*OA*_ under 100× and 50× as a function of the modulation frequency period (see Figure S3 in the Supporting Information). b) The OP–AP temperature difference ratio to AP temperature rise as a function of laser heating size for this work compared with previously reported data for 2D materials under CW laser heating.

To obtain *G*_int_, we construct the normalized Raman shift power coefficient Ω_exp_ = ψ_*FET*_/ψ_*CW*_ (see the Experimental Section) for optical and acoustic phonon temperatures separately. The Ω_*OP*_ is constructed using ψ_*OP*_ as measured in the experiment, which is proportional to the optical phonon temperature, whereas Ω_*AP*_is constructed for the corrected ψ_*AP*_. This treatment is meant to emphasize the impact of neglecting the OP–AP nonequilibrium on the measured interfacial thermal resistance (ITR or *R*′ as it appears in Equation (1)), which is the inverse of $G_{int}^{\prime }$. We then solve the heat equation (Equation (1)) numerically as a function of the ITR to construct the theoretical average temperature rise under the laser spot area for the transient heating (5 MHz modulation) relative to the steady state heating (denoted *Ω*). **Figure** **6**a illustrates the simulation results for the 293 K case. At each temperature (293 or 93 K), we use the corresponding temperature‐dependent thermophysical properties (thermal conductivity, heat capacity) as reported in the literature as input to our simulation.^[^ ^31^, ^56^ ^]^ The experimental Ω_*OP*_ and Ω_*AP*_ at 293 K are 0.881 and 0.837, respectively. These experimental results are mapped onto the solution, as shown in Figure 6a, to find the corresponding ITR. We find that using the optical phonon temperature underestimates the ITR (594 K m W^−1^), where it yields 69% of its intrinsic value (856 K m W^−1^) based on the acoustic phonon temperature. **Figure** **7**b compares the extracted intrinsic ITR values with those reported in the literature. At 293 K, our measured ITR lies within the range of previously published data for SWCNT/SiO_2_, indicating excellent agreement. At 93 K, our results are roughly 60% of the reported earlier but remain in the same overall order of magnitude.

To assess the temperature dependence of $G_{int}^{\prime }$ over a broader range of temperatures from such limited data, Al Keyyam and Wang^[^ ^57^ ^]^ proposed a pioneering universal theoretical model to describe interfacial thermal conductance named the EIM model. Unlike traditional models that assume an abrupt interface, the EIM treats the interface as a finite‐thickness layer with distinct properties. This concept is inspired by Guggenheim's interfacial model, where the interface is considered a separate medium with unique properties. In the EIM model, the interfacial thermal resistance, typically explained by phonon reflection, is replaced by conduction resistance through a defined layer of thickness *L*. The interfacial thermal conductance *G*_int_ is then simply given by *G*_int_ = *κ/L*, where *κ* is the equivalent thermal conductivity of the EIM which is dominated by localized oscillators that restrict the energy exchange to nearest neighbors (see the Experimental Section). The model accurately predicts the temperature dependence of *G*_int_ using only two measurements within the experimental uncertainty of standard measurement methods. This dramatically reduces the complexity and effort, particularly at challenging cryogenic conditions. Furthermore, normalizing the measured conductance data by the maximum achievable thermal conductance (*G*_int,max​_) predicted by the model and the temperature scale by the proposed interface characteristic temperature (*Θ*_int_​) (see the Experimental Section) reveals a previously unrecognized universal relationship across diverse interfaces where all literature data collapse into a single curve. The *Θ*_int_ encapsulates information about the strength of atomic bonding, the highest frequency the lattice can sustain, and the number of atoms per unit volume and is unique to the interfacial region. This universality mirrors the Debye normalization used for heat capacity in solids when normalized to the Debye temperature. More details can be found in the original publication.^[^ ^57^ ^]^

For van der Waals interfaces, the EIM is not intended to describe a physical interfacial layer of finite morphological thickness in the traditional sense. Rather, it represents a region that the van der Waals atomic interaction exists between atoms of SWCNTs and those of the substrate. Within this region, this van der Waals interaction is of high disorder, and features strong phonon localization. No distinct or gradual physical interlayer is expected to exist. This is a key distinction we emphasized in our earlier EIM work,^[^ ^57^ ^]^ where we discussed how van der Waals interfaces do not exhibit a measurable transition layer in terms of atomic structure, but they do present an energetically distinct region due to the unique chemical bonding responsible for localized phonon behavior.

In Figure 6c, we show that the EIM prediction based on our two measurements of *G*_int_ (assuming 1 nm contact width) is in excellent quantitative agreement with previous measurements done in our lab for assumed 1 nm contact width SWCNT bundle on SiO_2_ for a temperature range of 97–297 K^[^ ^30^ ^]^ and room temperature measurements.^[^ ^13^, ^29^ ^]^ The prediction is further found to match closely with the reported *G*_int_ of SWCNT thin films^[^ ^58^ ^]^ at a higher temperature range (300–450 K) further supporting the fitting results as shown in Figure 6b. We assert that the assumed 1 nm contact width is to estimate the order of magnitude per unit area and has no impact on the inferred *Θ*_int,_ which we find to be 1423 K for our interface. The measured *Θ*_int_ shown in Figure 6c is found to take an intermediate value between the Debye temperatures of SWCNT (≈2000 K)^[^ ^59^ ^]^ and SiO_2_ (470 K),^[^ ^60^ ^]^ consistent with previous findings in the original work for most interfaces found in literature.^[^ ^57^ ^]^ In Figure 6d, we further demonstrate the capability of the EIM prediction where we fit previously reported data^[^ ^57^ ^]^ by making use of only two measurements (shown in red solid stars) and mapping the rest of the experimental measurements (blue‐white triangles) onto the prediction of the EIM. One can clearly see the remarkable agreement between the two, which permits us to confidently use the EIM prediction as a basis for our results. A more comprehensive set of data can be found in the original work.^[^ ^57^ ^]^

As we mentioned earlier, the temperature rise ratio *ξ* is proportional to the lifetime of optical phonons *ξ*∝τ_ph_. Since the decay rate *τ*_ph_^−1^∝*T^n^* where *n* varies depending on the decay mechanism. For a 3‐phonon process, the exponent is not universal as it is shown to be material‐dependent, but becomes a linear function of temperature $\tau_{\mathrm{ph}}^{-1}\propto T$ at temperatures beyond the Debye temperature,^[^ ^61^ ^]^ whereas the relation is found to be quadratic (*n* = 2) for 4‐phonon decay.^[^ ^62^ ^]^ For our purposes, we keep *n* as a free parameter and fit *ξ* to have a *T* ^−*n*^ dependence (*ξ*∝*T* ^−*n*^). The fitting results shown in Figure 6e qualitatively resemble the dependence of phonon lifetime as plotted in the inset of Figure 4d.

The relation derived earlier *ξ* = Δ*T_OA_*/Δ*T_AP_* = *G*′_*int*_/(*G_OA_* · *A_c_*) is then utilized to infer the temperature‐dependent *G_OA_* as *G_OA_* = *G*′_*int*_/[(Δ*T_OA_*/Δ*T_AP_*) · *A_c_*]. First, we find the order of magnitude for the extracted *G_OA_* at room temperature 0.21 × 10^15^ W m^−3^ K^−1^ to have the same order of magnitude of that reported for MoSe_2_^[^ ^5^ ^]^ and MoS_2_^[^ ^19^ ^]^ but is one order of magnitude lower than predicted by Ruan and co‐workers for single‐layer graphene.^[^ ^24^ ^]^ Second, *G_OA_* is found to show a stronger temperature dependence, where it dies out more quickly than the interfacial phonon coupling across the interface. This can be understood by the exponential suppression of the population of optical phonons at low temperatures, unlike acoustic phonons, which can still exhibit considerable phonon population. We find *G*_*OA*,93*K*_/*G*_*OA*,293*K*_ to be about 5.8%, whereas *G*′_*int*,93*K*_/*G*′_*int*,293*K*_ is roughly 13.2%.

We emphasize that the fitting procedure has no impact on the reported *G_OA_* at 293 and 93 K, as those values are inferred directly from experimental data as measured. The fitting, nevertheless, predicts that the suppression of *G_OA_* gets more pronounced at lower temperatures. The *G*′_*int*_, on the other hand, is mainly governed by acoustic phonons, as mentioned earlier, and its temperature dependence can be assumed to take a *T* ^γ^at sufficiently low temperatures where *γ* depends on the dimensionality of the solid. This extra temperature dependence of *G_OA_* can also be understood by inspecting the relation *G_OA_* = *G*′_*int*_/[(Δ*T_OA_*/Δ*T_AP_*) · *A_c_*], where we proved earlier that ξ = Δ*T_OA_*/Δ*T_AP_* ∝*T* ^−*n*^ (*n* is a positive exponent) which yields an overall dependence of the form $G_{OA}\propto G_{int}^{\prime }T^{n}$. Earlier theoretical work shows that the phonon–phonon coupling factor is defined as *G*_p−p_ = *C*_ph_/τ_ph_^[^ ^24^ ^]^ which qualitatively yields a similar temperature dependence as asserted before when considering that τ_ph_∝*T* ^−*n*^ and that both $G_{int}^{\prime }$ and *C*_ph_ exhibit similar saturation behavior for sufficiently large temperatures (interface characteristic temperature *Θ*_int_ or Debye temperature of that particular phonon mode). We find that *G_OA_* does not saturate even for sufficiently high temperatures as shown in Figure 6e, unlike $G_{int}^{\prime }$ or $G_{int}$ which saturates as the temperature approaches the interface characteristic temperature *Θ*_int_.

We emphasize that usually two measurements are not sufficient to establish a firm temperature‐dependent relationship. The method used here to extract *G_OA_* relied on knowing the $G_{int}$. The extraction of a temperature‐dependent trend for $G_{int}$ from two measurements was only possible due to the recent theoretical model we developed – the EIM. Thus, conducting a systematic parametric study for detailed temperature dependence over a broad temperature range will be of great interest and could be considered in future work.

### 2.4 Laser Heating Size Effect and Literature Comparison

We repeat the experimental procedure outlined in Section 2.1 at an ambient temperature of 293 K under different laser spot radii (*r_o_*) for sample #2 (see Figure S2 in the Supporting Information for AFM scan). The laser spot size can be varied using different objective lenses. The experiment is conducted at 100× and 50× objective lenses, which corresponds to *r_o_* of about 0.37 and 0.76 µm, respectively, and the laser modulation frequency is varied from 10 to 25 MHz. Selected results under 100× are plotted in Figure S3 (Supporting Information). The same theoretical treatment outlined in Section 2.1 is used to extract ψ_0_ at the infinite frequency limit and quantify the optical–acoustic phonon nonequilibrium ψ_*OA*_. A summary of the measured and corrected *ψ* under 100× and 50× objective lenses is illustrated in **Figure** **7**a. The measured ψ_*CW*_, extracted ψ_0_, and ψ_*OA*_ are summarized in **Table** **2**. We find that ψ under 100× is larger than that under 50×, which can be understood by the higher thermal resistance for a smaller heating area.

**Table 2:** Summary of ψ values under different objective lenses.

Objective lens	|ψ_*CW*_| [cm^−1^ K^−1^]	|ψ_0_| [cm^−1^ K^−1^]	|ψ_*OA*_| [cm^−1^ K^−1^]
100×	0.183	0.109	0.034
50×	0.123	0.069	0.014

In Figure 7b, we compare Δ*T_OA_*/Δ*T_AP_* under different objective lenses. The results indicate that at a 0.37 µm laser spot radius (100× objective lens), Δ*T_OA_*/Δ*T_AP_* reaches nearly 23%. This ratio drops to ≈13% under 0.76 µm laser spot radius (50× objective). These results indicate that increasing the laser spot radius reduces the effect of optical–acoustic phonon nonequilibrium. This effect can be explained by the hot carrier diffusion and heat conduction out the laser heating region. The optical–acoustic phonon temperature difference is proportional to the local energy flux from OPs to APs. Under constant total laser energy input, we have $\Delta T_{OA}\propto {\left(\Delta r_{1}+r_{0}\right)}^{-2}$, where Δ*r*_1_​ is the radius extension beyond the laser heating spot *r*_0_ considering hot carrier diffusion from the laser heating region. This effect arises due to the negligible heat conduction by optical phonons, which rapidly transfer their energy to acoustic phonons before spatial redistribution.

On the other hand, $\Delta T_{AP}\propto {\left(\Delta r_{2}+r_{0}\right)}^{-2}$ where Δ*r*_2_ is the radius extension beyond the laser heating spot *r*_0_ due to diffusion of hot carriers and heat conduction, and we have Δ*r*_2_ > Δ*r*_1_. The smaller the laser spot size, the larger the influence of Δ*r*_1_ and Δ*r*_2_. For Δ*T_OA_*/Δ*T_AP_*, one can show that it scales with (Δ*r*_2_ + *r*_0_)^2^/(Δ*r*_1_ + *r*_0_)^2^. As the laser spot size decreases, this ratio increases. Consequently, the ratio Δ*T_OA_*/Δ*T_AP_* increases, explaining the observation in Figure 7b. At sufficiently large laser spots, the temperature difference between optical and acoustic branches becomes negligible, and the measured temperature rise in the Raman experiment can be safely assumed to represent Δ*T*_AP_. Even though Δ*T*_OA_ is a function of various optical–acoustic branch interactions rather than a singular value, we must emphasize that we report the average coupling between the optical and acoustic branches in the current study. In other words, our previous treatment assumes that all optical (acoustic) phonons are in thermal equilibrium within the same branch and share the same temperature.

To quantify the spatial extent of the in‐plane thermal transport and its impact on phonon nonequilibrium, we consider in‐plane heat transport in SWCNT bundle subjected to localized laser heating as follows. Because the heat spreads symmetrically along the length of the bundle, the center can be treated as the base of a very long fin, where the maximum temperature rise is attained at the center of the laser heating (the base). In this geometry, the spatial temperature distribution along the fin axis decays exponentially from the center as *e* ^−*mx*^ where *x* is the distance from the center of the heated region, and *m* encapsulates the balance between diffusive heat spreading and energy loss via the interfacial thermal conductance to the substrate which is defined as $m=\sqrt{{G^{\prime }}_{\mathrm{int}}/\kappa A_{c}}$ with units of inverse meters. Here, *κ* is the in‐plane thermal conductivity as measured previously in our lab,^[^ ^31^ ^]^ and *A*_c_ the solid cross‐sectional area as discussed earlier in Section 2.1. The corresponding characteristic length scale over which the temperature decays is *L*_th_ = 1/*m*, which we calculate to be around 0.8 µm at room temperature. For a 100× objective lens, the laser spot radius is 0.37 µm, which is much smaller than the characteristic length above. In this case, the deposited energy is confined to a subdiffusive region, which amplifies the ratio Δ*T_OA_*/Δ*T_AP_* as we observe in Figure 7b, mainly due to the participation of APs in carrying the heat outside the laser spot area at a greater rate under 100× as opposed to 50×. An earlier study by Sullivan et al.^[^ ^63^ ^]^ reported a similar observation where the phonon–phonon nonequilibrium exists and must be resolved for laser spot sizes smaller than the thermalization length for acoustic phonons.

We also compare our measured Δ*T_OA_*/Δ*T_AP_* with that of other suspended and supported 2D materials in Figure 7b. All the data confirm suppression of the OP–AP nonequilibrium effect as the laser spot radius increases. The absolute value of Δ*T_OA_*/Δ*T_AP_* varies from sample to sample. This can be explained as below. Under the same absorbed CW laser heating power (*E*), Δ*T*_OA_, to first order, is relatively unaffected by whether the material is suspended or supported. However, the subsequent thermal response of APs is highly sensitive to the presence of a substrate. In supported samples, APs can effectively dissipate heat through interfacial coupling to the substrate, leading to a reduced AP temperature rise Δ*T*_AP_. By contrast, suspended samples lack this interfacial dissipation channel, causing APs to have a higher Δ*T*_AP_. Consequently, Δ*T_OA_*/Δ*T_AP_* is generally larger for supported samples. Samples in refs. [5] and [15] are multilayered 2D materials, while the sample in ref. [19] is a monolayered 2D material. Δ*T*_OA_ in fact is proportional to the power density from OPs to APs, which is $E/(\pi r_{1}^{2}\Delta z)$ where Δ*z* is the sample thickness. Therefore, the monolayered sample in ref. [19] has a much higher Δ*T_OA_*/Δ*T_AP_*. For supported samples, this ratio is also greatly influenced by $G_{int}^{\prime }$ which is unique to each sample. This prevents us from making any quantitative comparison with previously reported data.

## 3 Conclusion

This work presents the first systematic experimental study of cryogenic optical–acoustic phonon nonequilibrium in SWCNTs employing FET‐Raman. Distinct optical–acoustic phonon temperature differences were observed, where the difference exceeds 75% of the lattice temperature rise at 93 K. This nonequilibrium is reduced substantially at room temperature due to intensified anharmonic phonon interactions. By proposing a rigorous theoretical argument and utilizing our recently developed EIM model, we quantified the temperature‐dependent optical–acoustic phonon coupling factor *G_OA_*, unveiling strong suppression at low temperatures that exceeds the suppression of the coupling of interfacial phonon modes, which sustain interfacial thermal conductance. Additionally, we measured the intrinsic interfacial thermal resistance based on the acoustic phonon temperature, which was underestimated if the optical phonon temperature is used. This warrants the need to account for phonon nonequilibrium to characterize thermal transport properties more accurately in conventional Raman thermometry. Our analysis further revealed that phonon nonequilibrium was sensitive to laser spot size, which significantly diminished with increased excitation area, aligning with previous studies in 2D materials. Detailed first‐principle calculations yet are needed to elaborate on the temperature‐dependent mode‐resolved phonon coupling factor beyond the effective one measured in this work. This work demonstrates a robust and precise experimental framework to probe phonon nonequilibrium in nanoscale materials, which opens new pathways to understand phonon transport that impacts thermal management, nanoscale energy conversion, and high‐performance electronic technologies.

## 4 Experimental Section

### Sample Preparation

SWCNTs were synthesized through an atmospheric pressure chemical vapor deposition method using sulfur, ferrocene, and xylene as the precursors. A nickel foil was positioned downstream in a quartz tube inside the chemical vapor deposition furnace, where the temperature gradually increased to 1160 °C under a flow of argon gas. The precursor mixture of sulfur and ferrocene dissolved in xylene was introduced upstream, and the carrier gas was switched to a mixture of argon and hydrogen. The gas flow rates were carefully regulated to optimize the conditions for growth. The duration of the reaction was adjusted to control the thickness of the resulting SWCNT layers. Aligned SWCNT arrays were formed by transferring the SWCNT film onto a 300 nm SiO_2_ layer on silicon. A razor blade was pressed against the SWCNT film, which was then moved in a single direction across the substrate to align the SWCNTs in the direction of the movement. To ensure uniform force distribution, a nylon filter soaked in ethanol was wrapped around the razor blade, which was used as a cushioning layer.^[^ ^64^ ^]^

### FET‐Raman

The FET‐Raman technique used in this work incorporated multienergy transport states under 50× and 100× objective lenses. For each case, a CW laser and a square wave amplitude‐modulated laser were employed with five different frequencies (5, 10, 15, 20, 25 MHz) and under two different ambient temperatures (93 and 293 K). The laser photon energy 2.33 eV, which corresponded to 532 nm wavelength, was chosen to facilitate a stronger Raman signal. Laser power (*P*) was systematically varied for each heating scenario using a LabVIEW‐controlled automated neutral‐density filter (model FW212CNEB). The laser power range was carefully chosen to generate a clear Raman signal and observable redshift while ensuring the sample remained undamaged. For Raman spectra acquisition, a HORIBA‐iHR550 imaging spectrometer and a confocal microscope were utilized. To modulate the amplitude of the CW laser into square‐wave pulses, an electro‐optic modulator (model 350‐160) was employed, with pulse shape and timing precisely controlled by a function generator (AFG31051), which allowed for pulses with specific laser‐on durations. The *G* band (≈1586 cm⁻¹) was chosen to serve as the temperature probe due to its strong response and high signal‐to‐noise ratio. To accurately determine the *G* band wavenumber (*ω*), a Gaussian fitting algorithm was used. By plotting the *ω* against laser power, the Raman shift power coefficient (ψ = ∂ω/∂*P*) was calculated, which was proportional to the sample's average temperature increase per 1 mW of incident laser power within the heated region. The normalized Raman shift power coefficient Ω_exp_ = ψ_*FET*_/ψ_*CW*_ was then calculated, which represented the average temperature rise in the transient state relative to the steady‐state heating. Since both ψ_*FET*_ and ψ_*CW*_ depended on the laser absorption and Raman temperature coefficients, the ratio effectively eliminated such dependencies to enhance accuracy. This normalization ensured that the ratio was governed solely by the thermal properties of the SWCNT bundle and its interaction with the substrate. The entire experimental procedures were performed repetitively under the different desired experimental setups mentioned earlier (objective lens, modulation frequencies, stage ambient temperatures). A more detailed description of the physics of the experiment could be found in the previous work.^[^ ^22^, ^29^, ^65^ ^]^

### EIM Model

Al Keyyam and Wang^[^ ^57^ ^]^ introduced a novel universal physics model for understanding and predicting interfacial thermal conductance through the development of what was termed the EIM, in which a finite‐thickness region (of thickness *L*) was introduced. The EIM possessed distinct properties governed by the interface characteristic temperature (*Θ*_int_), which was distinct from the Debye temperatures of adjacent materials. The interfacial thermal resistance traditionally attributed to phonon scattering was now redefined as a conduction resistance within the EIM sustained via special phonons termed interfacial phonons. Due to inherent structural irregularities in the EIM, since it was comprised of mixed materials, the model was built upon the seminal work of Cahill et al.^[^ ^66^ ^]^ on minimum thermal conductivity in disordered solids, which was an extension of Einstein's theory of thermal conductivity,^[^ ^67^ ^]^ where localized oscillators effectively sustained thermal transport. The EIM model was validated across a wide range of interfaces of different types and temperature ranges and was demonstrated to accurately predict interfacial thermal conductance from minimal data as few as two measurements. The thermal conductivity in Cahill's work was defined as

(3) $$\kappa ={\left(\frac{\pi }{6}\right)}^{1/3}k_{B}n^{2/3}\sum_{i}v_{i}{\left(\frac{T}{\Theta_{i}}\right)}^{2}\int_{0}^{\Theta_{i}/T}\frac{x^{3}e^{x}}{{\left(e^{x}-1\right)}^{2}}dx$$

The conditions were relaxed and it was assumed that the group velocity of the oscillators was independent of their polarization; hence, the sum could be reduced as $\sum_{i}v_{i}=3v_{\mathrm{avg},\mathrm{int}}$, where *i* is the index to sum over the three polarizations. *v*_avg,int_ and *L* were then combined into a single parameter termed the energy carrier transfer time defined as τ = *L*/*v*_avg,int_, which was the time taken for the energy carrier to transfer through the EIM. The final expression of interfacial thermal conductance (*G*) was then defined as *G* = *κ*/*L*

(4) $$G=\frac{3}{\tau }{\left(\frac{\pi }{6}\right)}^{1/3}k_{B}n^{2/3}{\left(\frac{T}{\Theta_{int}}\right)}^{2}\int_{0}^{\Theta_{int}/T}\frac{x^{3}e^{x}}{{\left(e^{x}-1\right)}^{2}}dx$$

Here, *k*_B_ is the Boltzmann constant, *n* is the number of atoms per unit volume, and is taken to be the harmonic average of the adjoining materials that constituted the interface, *Θ*_int_ is termed as the interface characteristic temperature, which shared a similar definition as the Debye temperature but is exclusive for the interface: Θ_int_ = *v*_avg,int_(ℏ/*k*_B_)(6π^2^ *n*)^1/3^, where ℏ is the reduced Planck's constant and *v*_avg,int_ is the group velocity of heat carriers. A more detailed discussion could be found in the original work.^[^ ^57^ ^]^

## Conflict of Interest

The authors declare no conflict of interest.

## Supporting information



Supporting Information

## Data Availability

The data that support the findings of this study are available from the corresponding author upon reasonable request.

## References

[1] H. Li , J. C. Grossman , Adv. Sci. 2017, 4, 1600467. 10.1002/advs.201600467 PMC5566246 28852610

[2] X. Wei , S. Li , W. Wang , X. Zhang , W. Zhou , S. Xie , H. Liu , Adv. Sci. 2022, 9, 2200054. 10.1002/advs.202200054 PMC9108629 35293698

[3] J. Zhu , R. Li , X. Tan , F. Feng , Q. Sun , P. Song , M. Hong , R. Ang , Adv. Funct. Mater. 2025, 35, 2417260.

[4] Y. D. Kim , H. Kim , Y. Cho , J. H. Ryoo , C.‐H. Park , P. Kim , Y. S. Kim , S. Lee , Y. Li , S.‐N. Park , Y. Shim Yoo , D. Yoon , V. E. Dorgan , E. Pop , T. F. Heinz , J. Hone , S.‐H. Chun , H. Cheong , S. W. Lee , M.‐H. Bae , Y. D. Park , Nat. Nanotechnol. 2015, 10, 676. 26076467 10.1038/nnano.2015.118

[5] R. Wang , H. Zobeiri , Y. Xie , X. Wang , X. Zhang , Y. Yue , Adv. Sci. 2020, 7, 2000097. 10.1002/advs.202000097 PMC7341092 32670758

[6] J. Xu , X. Huang , Y. Sheng , Q. Sun , H. Zhang , H. Bao , Y. Yue , Adv. Sci. 2025, 12, 2411040. 10.1002/advs.202411040 PMC11924035 39854132

[7] Q. Weng , L. Yang , Z. An , P. Chen , A. Tzalenchuk , W. Lu , S. Komiyama , Nat. Commun. 2021, 12, 4752. 34362908 10.1038/s41467-021-25094-5 PMC8346506

[8] F. Sekiguchi , H. Hirori , G. Yumoto , A. Shimazaki , T. Nakamura , A. Wakamiya , Y. Kanemitsu , Phys. Rev. Lett. 2021, 126, 077401. 33666485 10.1103/PhysRevLett.126.077401

[9] T. Faber , L. Filipovic , L. J. A. Koster , J. Phys. Chem. Lett. 2024, 15, 12601. 39681507 10.1021/acs.jpclett.4c03133 PMC11684017

[10] J. Yang , X. Wen , H. Xia , R. Sheng , Q. Ma , J. Kim , P. Tapping , T. Harada , T. W. Kee , F. Huang , Y.‐B. Cheng , M. Green , A. Ho‐Baillie , S. Huang , S. Shrestha , R. Patterson , G. Conibeer , Nat. Commun. 2017, 8, 14120. 28106061 10.1038/ncomms14120 PMC5263885

[11] E. Pop , Nano Res. 2010, 3, 147.

[12] M. S. Dresselhaus , P. C. Eklund , Adv. Phys. 2000, 49, 705.

[13] I. Al Keyyam , M. Rahbar , E. Shi , B. Li , T. Wang , X. Wang , J. Phys. Chem. C 2024, 128, 1505.

[14] D. Lee , J. Lee , W. Kim , Y. Suh , J. Park , S. Kim , Y. Kim , S. Kwon , S. Jeong , Adv. Sci. 2024, 11, 2308915. 10.1002/advs.202308915 PMC11348070 38932669

[15] M. Rahbar , I. Al Keyyam , J. Liu , X. Wang , Opt. Lett. 2024, 49, 4971. 39208011 10.1364/OL.532999

[16] S. Sullivan , K. Olsson , A. Weathers , E. Li , L. Shi , in APS March Meeting Abstracts, American Physical Society, New Orleans, LA 2017, p. H34.007.

[17] S. Xu , N. Hunter , H. Zobeiri , H. Lin , W. Cheng , X. Wang , Mater. Today Phys. 2022, 27, 100816.

[18] H. Zobeiri , N. Hunter , R. Wang , T. Wang , X. Wang , Adv. Sci. 2021, 8, 2004712. 10.1002/advs.202004712 PMC8224447 34194932

[19] N. Hunter , N. Azam , H. Zobeiri , N. Van Velson , M. Mahjouri‐Samani , X. Wang , Adv. Mater. Interfaces 2022, 9, 2102059.

[20] S. Hu , C. Zhao , X. Gu , Int. J. Therm. Sci. 2024, 196, 108725.

[21] Y. Zhu , T. Ye , H. Wen , R. Xu , Y. Zhong , G. Lin , D. Liang , W. Cai , D. Yu , W. Lin , Adv. Funct. Mater. 2024, 34, 2407333.

[22] J. Liu , I. Al Keyyam , Y. Xie , X. Wang , Surf. Sci. Technol. 2024, 2, 8.

[23] A. K. Vallabhaneni , D. Singh , H. Bao , J. Murthy , X. Ruan , Phys. Rev. B 2016, 93, 125432.

[24] Z. Lu , A. Vallabhaneni , B. Cao , X. Ruan , Phys. Rev. B 2018, 98, 134309.

[25] N. Bonini , M. Lazzeri , N. Marzari , F. Mauri , Phys. Rev. Lett. 2007, 99, 176802. 17995357 10.1103/PhysRevLett.99.176802

[26] İ. Doğan , R. Gresback , T. Nozaki , M. C. M. van de Sanden , Sci. Rep. 2016, 6, 29508. 27389331 10.1038/srep29508 PMC4937401

[27] N. Gaponik , Y. P. Rakovich , M. Gerlach , J. F. Donegan , D. Savateeva , A. L. Rogach , Nanoscale Res. Lett. 2006, 1, 68.

[28] C. Li , S. Xu , Y. Yue , B. Yang , X. Wang , Carbon 2016, 103, 101.

[29] I. Al Keyyam , B. Li , T. Wang , C. Deng , X. Wang , Carbon 2025, 233, 119906.

[30] I. Al Keyyam , M. Rahbar , N. Hunter , B. Li , T. Wang , E. Shi , X. Wang , Int. J. Heat Mass Transfer 2024, 226, 125513.

[31] M. Rahbar , B. Li , N. Hunter , I. Al Keyyam , T. Wang , E. Shi , X. Wang , Cell Rep. Phys. Sci. 2023, 4, 101688.

[32] Z. Tian , J. Garg , K. Esfarjani , T. Shiga , J. Shiomi , G. Chen , Phys. Rev. B 2012, 85, 184303.

[33] P. Tengdin , W. You , C. Chen , X. Shi , D. Zusin , Y. Zhang , C. Gentry , A. Blonsky , M. Keller , P. M. Oppeneer , H. C. Kapteyn , Z. Tao , M. M. Murnane , Sci. Adv. 2018, 4, aap9744. 10.1126/sciadv.aap9744 PMC5834307 29511738

[34] W. You , P. Tengdin , C. Chen , X. Shi , D. Zusin , Y. Zhang , C. Gentry , A. Blonsky , M. Keller , P. M. Oppeneer , H. Kapteyn , Z. Tao , M. Murnane , Phys. Rev. Lett. 2018, 121, 077204. 30169091 10.1103/PhysRevLett.121.077204

[35] C. Rani , M. Tanwar , T. Ghosh , S. Kandpal , S. K. Saxena , R. Kumar , Phys. Rep. 2023, 1037, 1.

[36] T. M. Gibbons , M. B. Bebek , B. Kang , C. M. Stanley , S. K. Estreicher , J. Appl. Phys. 2015, 118, 085103.

[37] P. Anees , M. C. Valsakumar , B. K. Panigrahi , Phys. Chem. Chem. Phys. 2016, 18, 2672. 26705543 10.1039/c5cp06111c

[38] M. A. Al‐Nimr , S. A. Masoud , J. Heat Transfer 1997, 119, 188.

[39] M. A. Al‐Nimr , Int. J. Thermophys. 1997, 18, 1257.

[40] K. Ramadan , W. R. Tyfour , M. A. Al‐Nimr , J. Heat Transfer 2009, 131, 111301.

[41] J. L. Hostetler , A. N. Smith , D. M. Czajkowsky , P. M. Norris , Appl. Opt. 1999, 38, 3614. 18319965 10.1364/ao.38.003614

[42] M. Balkanski , R. F. Wallis , E. Haro , Phys. Rev. B 1983, 28, 1928.

[43] T.‐M. Liu , S.‐Z. Sun , C.‐F. Chang , C.‐C. Pan , G.‐T. Chen , J.‐I. Chyi , V. Gusev , C.‐K. Sun , Appl. Phys. Lett. 2007, 90, 041902.

[44] I. Chatzakis , H. Yan , D. Song , S. Berciaud , T. F. Heinz , Phys. Rev. B 2011, 83, 205411.

[45] M. Steiner , M. Freitag , V. Perebeinos , J. C. Tsang , J. P. Small , M. Kinoshita , D. Yuan , J. Liu , P. Avouris , Nat. Nanotechnol. 2009, 4, 320. 19421219 10.1038/nnano.2009.22

[46] P. G. Klemens , Phys. Rev. 1966, 148, 845.

[47] S. Katsiaounis , N. Delikoukos , A. Michail , J. Parthenios , K. Papagelis , Carbon 2023, 215, 118449.

[48] Y. Xie , Z. Xu , S. Xu , Z. Cheng , N. Hashemi , C. Deng , X. Wang , Nanoscale 2015, 7, 10101. 25981826 10.1039/c5nr02012c

[49] Z. Han , X. Yang , S. E. Sullivan , T. Feng , L. Shi , W. Li , X. Ruan , Phys. Rev. Lett. 2022, 128, 045901. 35148139 10.1103/PhysRevLett.128.045901

[50] T. Feng , X. Ruan , Phys. Rev. B 2018, 97, 045202.

[51] Y. Liu , Y. Shi , W. Zhou , W. Shi , W. Dang , X. Li , B. Liang , Opt. Laser Technol. 2021, 139, 106960.

[52] T. Beechem , S. Graham , J. Appl. Phys. 2008, 103, 093507.

[53] J. F. Cardenas , A. Gromov , Nanotechnology 2009, 20, 465703. 19843989 10.1088/0957-4484/20/46/465703

[54] J. Wang , Z. Wang , K. Yang , N. Chen , J. Ni , J. Song , Q. Li , F. Sun , Y. Liu , T. Fan , Adv. Funct. Mater. 2022, 32, 2206545.

[55] Z.‐Y. Ong , E. Pop , J. Shiomi , Phys. Rev. B 2011, 84, 165418.

[56] W. DeSorbo , W. W. Tyler , J. Chem. Phys. 1953, 21, 1660.

[57] I. Al Keyyam , X. Wang , Mater. Today Phys. 2024, 46, 101516.

[58] A. Duzynska , A. Taube , K. P. Korona , J. Judek , M. Zdrojek , Appl. Phys. Lett. 2015, 106, 183108.

[59] Z. Yao , C. L. Kane , C. Dekker , Phys. Rev. Lett. 2000, 84, 2941. 11018981 10.1103/PhysRevLett.84.2941

[60] W. Zhu , G. Zheng , S. Cao , H. He , Sci. Rep. 2018, 8, 10537. 30002417 10.1038/s41598-018-28925-6 PMC6043512

[61] G. L. Guthrie , Phys. Rev. 1966, 152, 801.

[62] T. Feng , X. Ruan , Phys. Rev. B 2016, 93, 045202.

[63] S. Sullivan , A. Vallabhaneni , I. Kholmanov , X. Ruan , J. Murthy , L. Shi , Nano Lett. 2017, 17, 2049. 28218545 10.1021/acs.nanolett.7b00110

[64] E. Shi , H. Li , L. Yang , J. Hou , Y. Li , L. Li , A. Cao , Y. Fang , Adv. Mater. 2015, 27, 682. 25607917 10.1002/adma.201403722

[65] H. Zobeiri , R. Wang , T. Wang , H. Lin , C. Deng , X. Wang , Int. J. Heat Mass Transfer 2019, 133, 1074.

[66] D. G. Cahill , S. K. Watson , R. O. Pohl , Phys. Rev. B 1992, 46, 6131. 10.1103/physrevb.46.6131 10002297

[67] A. Einstein , Ann. Phys. 1911, 340, 679.

