# Supplementary material for: Direct Observation of Suppressed Optical–Acoustic Phonon Energy Coupling in Supported SWCNT at Cryogenic Temperatures
Source: Adv Sci (Weinh). 2025 Jul 3;12(37):e09005. doi: 10.1002/advs.202509005 (PMC12499449; doi:10.1002/advs.202509005)
Supplement: Supplementary file 1 — Supporting Information [file ADVS-12-e09005-s001.docx]

**Supporting Information for**

**Direct Observation of Suppressed Optical-Acoustic Phonon Energy Coupling in Supported SWCNT at Cryogenic Temperatures**

Ibrahim Al Keyyam^†^, Yu Hua^†^, Baini Li, Tianyu Wang, Cheng Deng^*^, Xinwei Wang^[[1]](#footnote-2)^

Ibrahim Al Keyyam, Yu Hua, Prof. Xinwei Wang

Department of Mechanical Engineering

Iowa State University

Ames, IA 50011, USA

Email: xwang3@iastate.edu

Baini Li, Dr. Tianyu Wang

Research Center for Industries of the Future, Key Laboratory of 3D Micro/Nano Fabrication and Characterization of Zhejiang Province, School of Engineering

Westlake University

Hangzhou, 310024, P. R. China

Prof. Cheng Deng

College of Mechatronics Engineering

Guangdong Polytechnic Normal University

Guangzhou, 510635, P. R. China

Email: dengcheng@gpnu.edu.cn

**Section S1. Temperature Control Stage**

**Section S2. Atomic Force Microscope Image of Sample #2**

**Section S3. Representative Raman Experimental Measurements of Sample #2**

**S1. Temperature Control Stage**


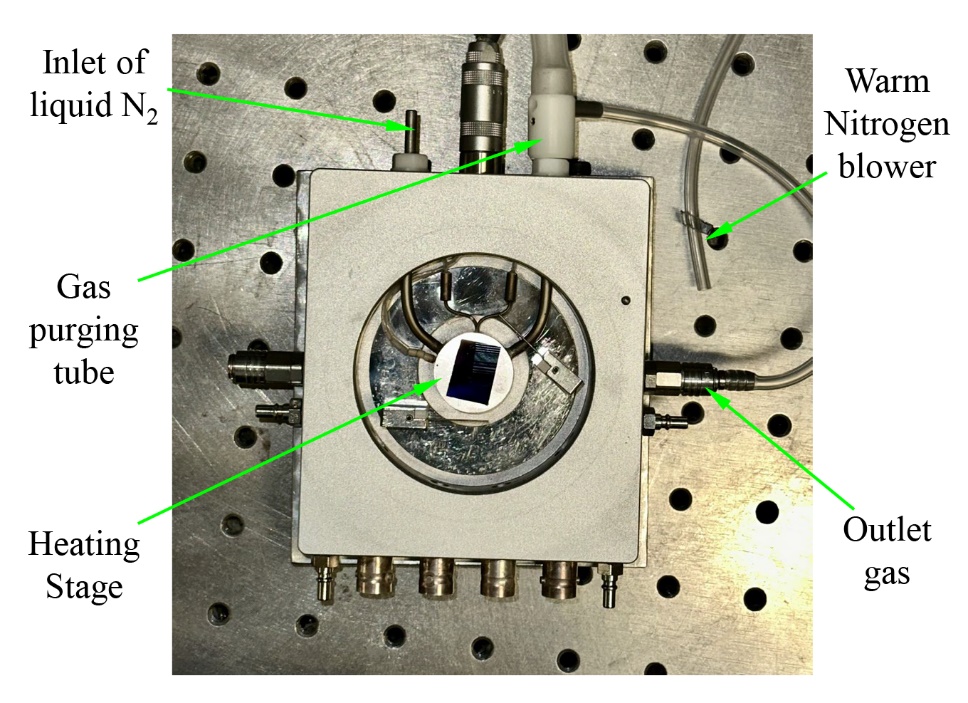


**Figure S1.** Temperature control stage LINKAM Scientific HFS600E-PB4. The stage employs software-controlled integrated resistive electrical heating and liquid nitrogen cooling. A high-precision temperature sensor continuously monitors stage temperature, supplies immediate feedback to the software to adjust heating or cooling based on deviations from the setpoint, and achieves temperature stability with a resolution of 0.01°C. The warm nitrogen blower utilizes warm recycled nitrogen gas to prevent water condensation on the lid window.

**S2. Atomic Force Microscope Image of Sample #2**


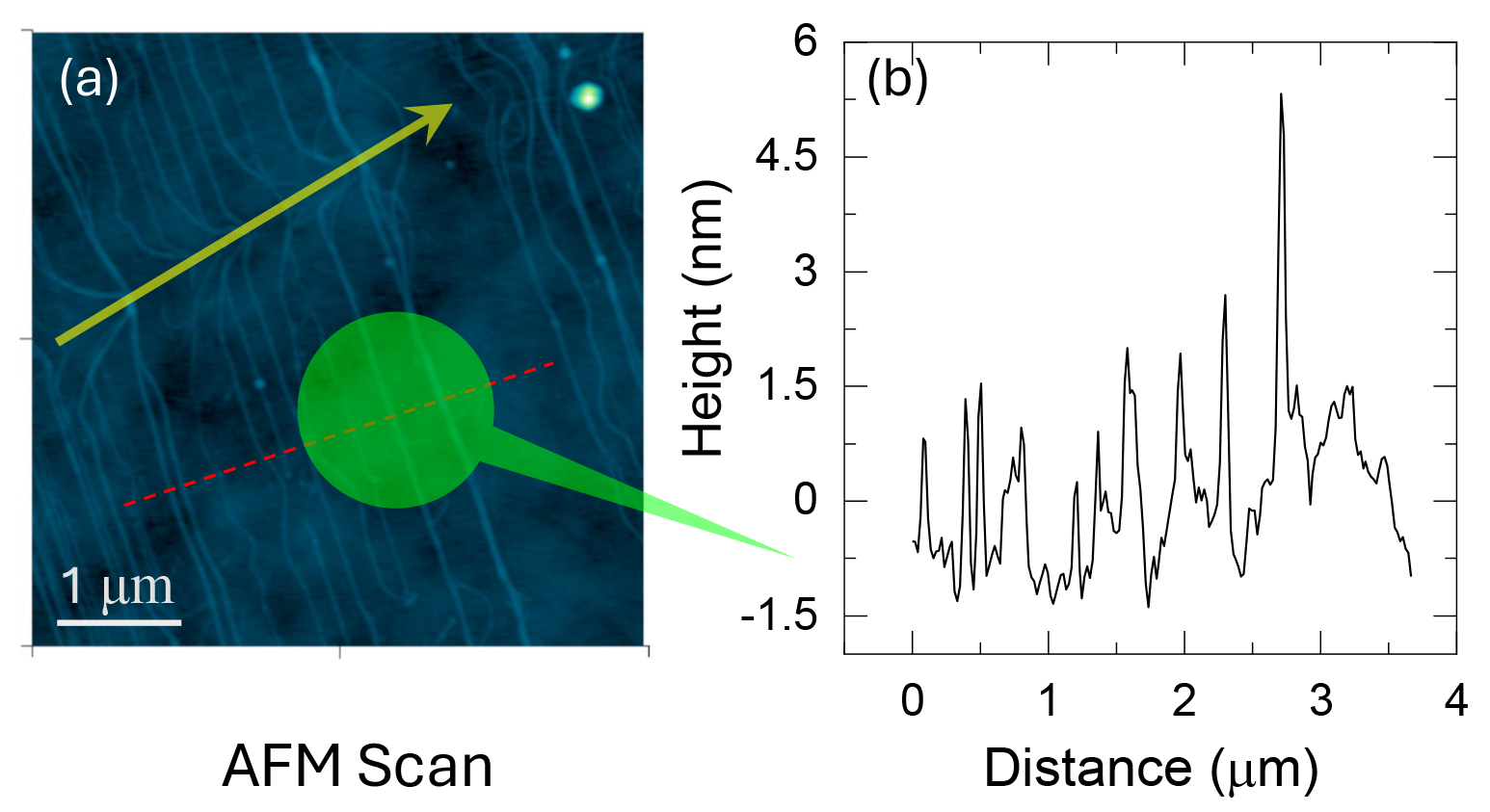


**Figure S2.** (a) AFM scan of sample #2. We observe an abnormal disruption for the alignment of the bundles shown in the yellow arrow. It is plausible that this happened by the tip while scanning before capturing the final image shown of the AFM scan. (b) Height measurements of the bundles which span few nanometers. Note that for the analysis of sample #2 (see Section 2.4 in the manuscript), we did not perform any quantitative measurement that requires the size/mass of the bundle.

**S3. Representative Raman Experimental Measurements of Sample #2**


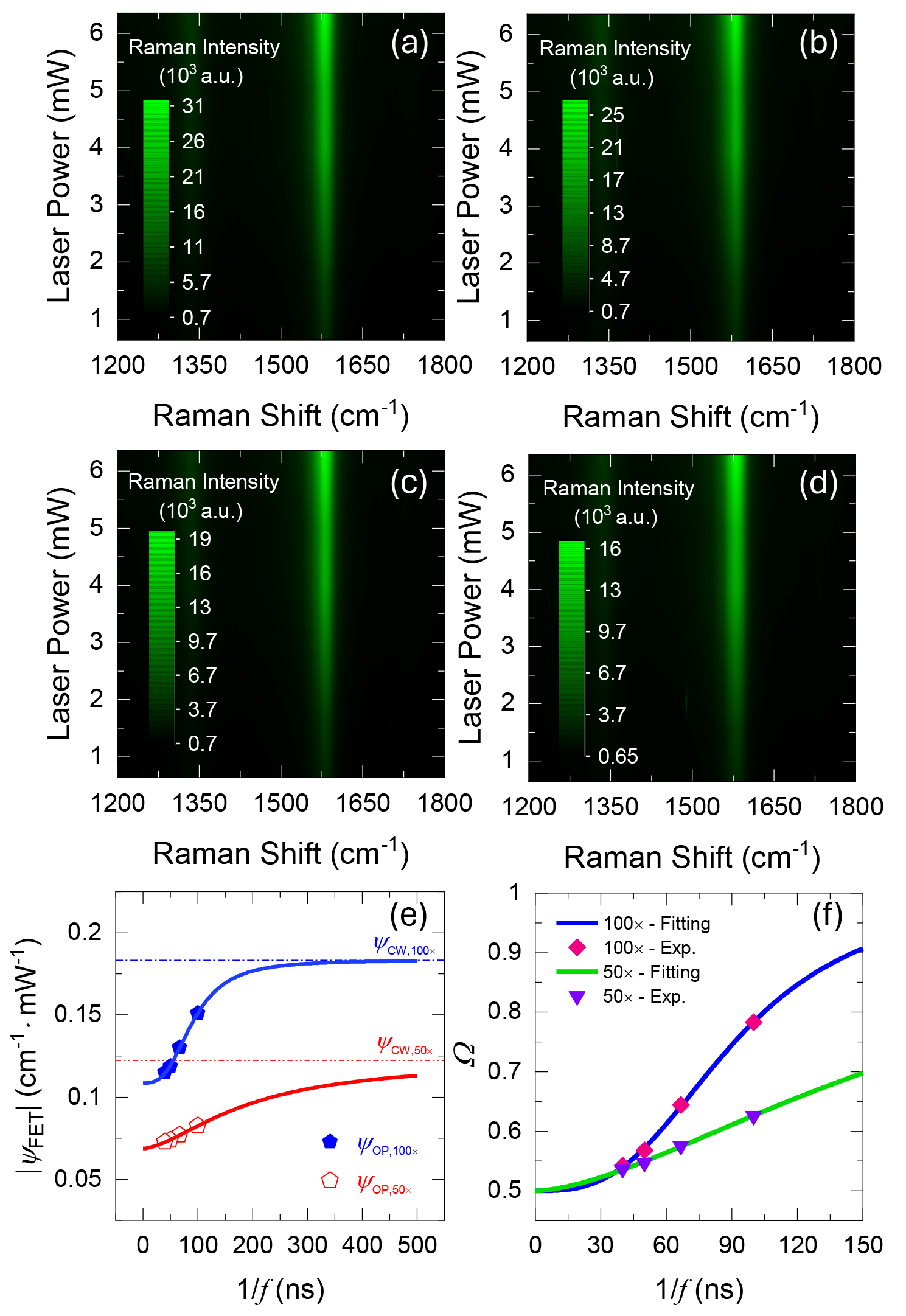


**Figure S3.** The Raman intensity and wavenumber variation with laser power under 100× objective lens for (a) CW heating. (b) 15 MHz. (c) 20 MHz. (d) 25 MHz. (e) The experimental measured $\psi_{OP}$ under 100× and 50× with their fitting that approaches the $\psi_{CW}$ at sufficiently large heating time. (f) The normalized acoustic phonon temperature rise for transient heating relative to steady state heating for 50× and 100× objective lens. The ratio approaches 0.5 at the infinite frequency limit, as discussed in Section 2.1.

1. Corresponding authors: dengcheng@gpnu.edu.cn (C. Deng), xwang3@iastate.edu (X. Wang) (001-515-294-8023).

   ^†^ Equal contribution authors. [↑](#footnote-ref-2)
